# Prospective, longitudinal analysis of the gut microbiome in patients with locally advanced rectal cancer predicts response to neoadjuvant concurrent chemoradiotherapy

**DOI:** 10.1186/s12967-023-04054-1

**Published:** 2023-03-26

**Authors:** Yi Sun, Xiang Zhang, Chuandi Jin, Kaile Yue, Dashuang Sheng, Tao Zhang, Xue Dou, Jing Liu, Hongbiao Jing, Lei Zhang, Jinbo Yue

**Affiliations:** 1 Department of Radiation Oncology, Shandong Cancer Hospital and Institute, Shandong First Medical University and Shandong Academy of Medical Sciences, Jinan, China; 2 The Comprehensive Cancer Centre of Nanjing Drum Tower Hospital, The Affiliated Hospital of Nanjing University Medical School, Nanjing, China; 3 Department of Biostatistics, School of Public Health, Cheeloo College of Medicine, Shandong University, Jinan, China; 4 Microbiome-X, National Institute of Health Data Science of China & Institute for Medical Dataology, Cheeloo College of Medicine, Shandong University, Jinan, China; 5 Department of Pathology, Shandong Cancer Hospital and Institute, Shandong First Medical University and Shandong Academy of Medical Sciences, Jinan, China; 6 State Key Laboratory of Microbial Technology, Shandong University, Qingdao, China

**Keywords:** Gut microbiota, Rectal cancer, Neoadjuvant chemoradiotherapy, Immunomodulatory protein, Microbiome profile, *Intestinimonas*

## Abstract

**Background:**

Neoadjuvant concurrent chemoradiotherapy (nCCRT) is a standard treatment for locally advanced rectal cancer (LARC). The gut microbiome may be reshaped by radiotherapy through its effects on microbial composition, mucosal immunity, and the systemic immune system. We sought to clarify dynamic, longitudinal changes in the gut microbiome and blood immunomodulators throughout nCCRT and to explore the relationship of such changes with outcomes after nCCRT.

**Methods:**

A total of 39 patients with LARC were recruited for this study. Fecal samples and peripheral blood samples were collected from all 39 patients before nCCRT, during nCCRT (at week 3), and after nCCRT (at week 5). The gut microbiota and the microbial community structure were analyzed by 16S rRNA sequencing of the V3–V4 region. Levels of blood immunomodulatory proteins were measured with a Millipore HCKPMAG-11 K kit and Luminex 200 platform (Luminex, USA).

**Results:**

Cross-sectional and longitudinal analyses revealed that the gut microbiome profile and enterotype exhibited characteristic variations that could distinguish patients with good response (AJCC TRG classification 0–1) vs poor response (TRG 2–3) to nCCRT. Sparse partial least squares regression and canonical correspondence analyses showed multivariate associations between specific microbial taxa, host immunomodulatory proteins, immune cells, and outcomes after nCCRT. An integrated model consisting of baseline *Clostridium *sensu stricto* 1* levels, fold changes in *Intestinimonas*, blood levels of the herpesvirus entry mediator (HVEM/CD270), and lymphocyte counts could predict good vs poor outcome after nCCRT [area under the receiver-operating characteristics curve (AUC)= 0.821; area under the precision-recall curve [AUPR] = 0.911].

**Conclusions:**

Our results showed that longitudinal variations in specific gut taxa, associated host immune cells, and immunomodulatory proteins before and during nCCRT could be useful for early predictions of the efficacy of nCCRT, which could guide the choice of individualized treatment for patients with LARC.

**Supplementary Information:**

The online version contains supplementary material available at 10.1186/s12967-023-04054-1.

## Introduction

Colorectal cancer is the fifth most commonly diagnosed cancer and the fifth leading cause of cancer-related mortality in China [1]. The standard of care for patients with locally advanced rectal cancer (LARC) is neoadjuvant concurrent chemoradiotherapy (nCCRT), typically followed by total mesorectal excision [2]. Response is generally assessed by pathologic determination of primary tumor regression grade (TRG), which is based on the amount of residual tumor cells and the extent of desmoplastic (fibrotic) response in biopsy samples obtained after nCCRT [3]. TRG is considered an independent prognostic factor in rectal cancer and has been linked with the risk of developing distant metastases and with disease-free survival [4, 5]. Novel markers in addition to TRG with which to monitor treatment response may prove to be useful for identifying such patients.

One such biomarker of response after nCCRT may be aspects of the gut microbiome, which has been implicated in antitumor responses to immunotherapy and chemotherapy and characterized as one of four new hallmarks of cancer [6, 7]. The intestinal microbiome has vital roles in the maintenance of its mucosal barrier and homeostasis [8]. Gut microbes participate in the regulation of oncogenesis, tumor progression, and therapeutic effects in gastrointestinal tumors by producing metabolites and toxins that affect cell proliferation, modulate inflammation and immunity, and induce DNA damage [9, 10]. The diversity and composition of the gut microbiome also influence the toxicity and effectiveness of chemotherapy, radiotherapy, and immune-checkpoint blockers; conversely, chemotherapy, radiotherapy, and immunotherapy have been shown to remodel the composition of the gut microbiome in preclinical models and clinical trials [11, 12]. However, little is known of the relationship between the gut microbiome and pathologic response among patients with LARC undergoing nCCRT. In the current study, we collected feces and peripheral blood samples from patients with LARC at three timepoints: before, during, and after nCCRT, and the objective of this study was to evaluate correlations between longitudinal changes in the gut microbiota and pathologic response throughout the course of nCCRT.

## Methods

### Participants and sample collection

Participants were 39 patients with newly diagnosed LARC who were enrolled at the Shandong Cancer Hospital and Institute from February 2018 to June 2019, and all patients provided written informed consent to participate. Exclusion criteria were use of adrenocortical hormones or other immunosuppressive drugs within the past 6 months, or use of systemic antibiotics or probiotics during the 3 months before nCCRT, during nCCRT, or before surgery. None of the enrolled patients had a history of inflammatory bowel disease or irritable bowel syndrome. All patients received 50 Gy of radiotherapy in 25 fractions in conjunction with oral capecitabine (1650 mg/m^2^/day, 5 days/week). The multidisciplinary team at the Shandong cancer hospital justified the use of induction chemotherapy for each patient. 21 patients considered at high risk of metastasis (N2 stage, bulky tumor, lateral lymph node metastasis or extramural vascular invasion) received induction chemotherapy (a single cycle of oral capecitabine at 1000 mg/m^2^ twice daily for 14 days and intravenous oxaliplatin at 130 mg/m^2^ on the first day) before nCCRT at the discretion of the treating physician.

We used a semi-quantitative method to assess the pathological grade of primary tumor regression by measuring the amount of residual tumor cells as well as the desmoplastic response. The American Joint Commission on Cancer (AJCC) TRG classification groups were as follows: TRG0, no residual tumor cells; TRG1, single cells or small groups of cells; TRG2, residual cancer with desmoplastic response; and TRG3, minimal evidence of tumor response. Patients were then grouped as good responders (TRG 0,1) or poor responders (TRG 2,3).

A total of 35 feces samples were collected from the 39 patients before nCCRT (good = 20, poor = 15), 31 during nCCRT (good = 15, poor = 16), and 32 after nCCRT. All enrolled patients had clinical T3-4N0-2M0 disease and received preoperative nCCRT followed by surgery. Samples of feces and peripheral blood were collected from the enrolled patients before nCCRT, during nCCRT (at week 3), and after nCCRT (at week 5). Pathological responses were determined by two independent pathologists who evaluated biopsy or surgical specimens according to the 8th edition of the AJCC TRG system [4]. Fecal samples were stored in stool collection tubes at – 80 °C before 16S rRNA gene sequencing. Fresh peripheral blood samples were centrifuged at 1200 rpm for 5 min and the upper layer was collected in Eppendorf centrifuge tubes, which were centrifuged further at 12,000 rpm for 15 min to produce the serum, which was extracted and stored at – 80 °C in preparation for testing.

### Immunomodulators

Levels of soluble immunomodulatory proteins were measured in serum samples obtained before, during, and after nCCRT with a Millipore HCKPMAG-11 K kit and Luminex 200 platform (Luminex, USA). Markers measured were CD27, CD28, TIM3, CD40, GITR, PD-1, CTLA4, CD80, CD86, PD-L1, inducible co-stimulator, tumor necrosis factor-alpha, interleukin (IL) -6, -10, and -1-alpha, interferon-gamma, and granulocyte–macrophage colony-stimulating factor. We also analyzed temporal changes in the platelet/lymphocyte ratio, neutrophil/lymphocyte ratio, and systemic immune-inflammation index (platelet count × neutrophil count/lymphocyte count). We conducted exploratory correlation analysis of the top 40 most abundant genera and these immunologic and inflammatory markers to provide hypotheses for subsequent clinical and basic research.

### DNA extraction and 16S rRNA gene sequencing

DNA was extracted from fecal samples collected before, during, and after nCCRT with a QIAamp DNA Stool Mini Kit (Qiagen, Germany). The V4 variable region of the 16S rRNA gene was amplified by PCR and the primer sequences were 515F (5′-GTGCCAGCMGCCGCGGTAA-3′) and 806R (5′-GGACTACHVGGGTWTCTAAT-3′) modified with specific barcodes. Sequencing libraries were generated with the Ion Plus Fragment Library Kit (48 rxns, Thermo Scientific), and the quality of the library was assessed with a Qubit 2.0 Fluorometer (Thermo Scientific). Purified PCR products were prepared for amplicon sequencing with an IonS5 TMXL platform (Thermo Fisher), and 400-bp single-end reads were generated.

### Raw data processing and analysis

Raw sequencing data were manipulated by using Quantitative Insights Into Microbial Ecology 2 (QIIME2, version 2020.2) [13]. Briefly, single-end reads were assigned to samples based on their unique barcode and truncated by cutting off the barcode and primer sequence. The demultiplexed reads were then denoised by using the q2-dada2 plugin [14] in QIIME2 for quality control (-p-trunc-len 0, -p-trim-length 250), chimera detection and removal [14], and generation of amplicon sequence variants (ASVs) and representative sequences. Taxonomy of the ASVs was assigned by using the SILVA database (version 138) [15] classifier with 99% similarity. Samples with fewer than 10 ASVs and representative sequences with a frequency of < 10 were removed. Finally, a total of 2606 ASVs were retained, with the number of reads ranging from 28,267 to 47,749.

### Batch effect correction

In this study, 16S rRNA gene sequencing of the 98 collected fecal samples was done separately at two different times, resulting in batch1 (n = 69) and batch2 (n = 29). This may have introduced a batch effect because several technicians ran the sequencing on different machines. To determine whether a batch effect was present in the microbiome data, we compared alpha diversity, beta diversity, and taxa between the two batches as follows. Alpha diversity was measured with the Observed, Shannon, InvSimpson, and Chao1 indexes, and rarefaction curves; beta diversity was assessed after normalization by centered log-ratio transformation (measured as the Bray–Curtis distance). Differences between the two batches were then compared by using the R package phyloseq (version 1.34.0) [16]. Linear discriminant analysis effect size (LEfSe) analysis was conducted to identify any differences in taxa between the two batches by using the *diff_analysis* function in the R package MicrobiotaProcess (version 1.2.0) [17, 18]. The ComBat method [19–21] was used to correct batch effects in microbiome data, and was done with the R package sva (version 3.38.0) [22] After batch effect correction, the alpha diversity, beta diversity and LEfSe analyses were run again to assess whether any batch effect had been successfully removed. All subsequent analyses were done with the corrected microbiome data.

### Associations between microbiome profiles and outcome after nCCRT

For each timepoint (before, during, and after nCCRT), alpha diversity (measured by the Observed, Shannon, InvSimpson and Chao1 index, rarefaction curve), beta diversity (measured by the Bray–Curtis distance), and differences in taxa (LEfSe analysis) were compared between the good- and poor-response groups. We used a paired and a longitudinal distance (pldist)-based approach [23, 24] to account for within-individual shifts of beta diversity in microbiome composition, and we then compared those compositional shifts between the good-response and poor-response groups over time. Two-part zero-inflated beta regression with random effects (ZIBR) models [25–27] were also used to test associations between microbial abundance over time (longitudinal analysis) and outcome after nCCRT. Alluvial diagrams displaying the longitudinal profiles of the top 20 abundant genera (after ignoring the “other” category) in both the good- and poor-response groups were generated by using the R package ggalluvial (version 0.12.3) [28]. To calculate the variation contributed by patient characteristics on the microbial profiles over time, we used a permutational multivariate analysis of variance (PERMANOVA) test (Adonis) with the R package vegan (version 2.5.7), and *P* values were generated. Finally, to determine whether receipt of induction chemotherapy before nCCRT affected the microbiome profiles, the alpha diversity, beta diversity, and LEfSe analysis results were compared between patients who received induction chemotherapy and those who did not with the same methods as described above.

### Community state types analysis

Partitioning-around-medoid (PAM) clustering based on a Jensen-Shannon distance of the microbial count data was used to assign samples to several community state types (CSTs) with the R package Cluster (version 2.0.6) [29], in which both gap statistic evaluation and silhouette width quality validation were used to determine cluster formation. Nonmetric multidimensional scaling analysis was further used to assess diversity among CSTs with the R package phyloseq (version 1.34.0) [16]. To assess consistency of CSTs before, during, and after nCCRT, inter-rater agreement (kappa) was calculated for the good- and poor-response groups. Significant differences in microbial communities in the CSTs were identified by LEfSe with the R package MicrobiotaProcess (version 1.2.0), which includes use of the false discovery rate (FDR) method to correct for multiple comparisons as a default. LEfSe was also used to identify microbial features whose abundance differed between the good- and poor-response groups. Transitions between CSTs over time were assessed for both the good- and poor-response groups.

### Multivariate analyses

Clustering analysis of immune cells and immunomodulatory proteins based on Spearman’s rank correlation was done with the R packages Hmisc (version 4.6.0) [30] and corrplot (version 0.90) [31]. We used multivariate analyses to explore the interrelationships between the microbiome profiles and immune cells and immunomodulatory proteins at each timepoint relative to nCCRT. First, the microbial community data matrix at each timepoint was calculated separately based on Bray–Curtis distance by using the R package phyloseq (version 1.34.0) [16]. Based on these matrices, potentially relevant immune factors (*P* ≤ 0.05) were then filtered by using the Adonis method with the R package vegan (version 2.5.7) for subsequent multivariate analyses [32].

The R package mixOmics (version 6.14.0) [33] was then used for sparse partial least squares (sPLS) regression analyses at each timepoint, with microbial community data matrices and the matrix of filtered immune factors, followed by hierarchical clustering analysis based on Pearson’s correlation; ASVs with an absolute correlation coefficient value of > 0.2 were retained for subsequent canonical correspondence analysis (CCpnA). Loading vectors of specific ASVs that exhibited high loading weights (> 0.1 or <  − 0.1) to the separation of clinical variables were also obtained and visualized with loading plots.

Next, we applied CcpnA to model the canonical relationship between ASV abundance and factors such as baseline patient characteristics and immune factors at each timepoint by using the R package vegan (version 2.5.7) [32].

### Predictive model construction

To assess whether combinations of microbes, immune cells, and immunomodulatory proteins could predict outcome after nCCRT, we identified potential candidates as follows. Candidate microbes were derived from the genera that differed between the good- and poor-response groups, as identified in cross-sectional analysis (LEfSe analysis before and during nCCRT) and longitudinal analysis (ZIBR analysis) as well as genera that contributed to microbial clustering in multivariate statistical analysis (sPLS before and during nCCRT). Candidate immune factors were identified by Adonis for factors measured before or during nCCRT that were significantly related to the variation in microbiome profiles. After patients were randomized 1:1 to a training set and an internal validation (test) set, candidate factors at each measurement time (before and during nCCRT) served as variables in a regularized regression approach to creating a least absolute shrinkage and selection operator (LASSO) model (adjusted for age, sex, body mass index [BMI] and induction chemotherapy, and including tenfold cross-validation, Alpha = 1, lambda = lambda.min, and TRG as classification variables) to develop the signature combinations that best predicted prognosis by using the R package glmnet (version 4.1). LASSO analysis was then conducted again with both baseline levels and log2-transformed fold-changes of candidate variables before vs during nCCRT. Receiver-operating characteristic (ROC) curve analysis (with the R package ROCR [v1.0–11]) was used to determine the best cut-off values. Model performance was assessed by analyzing both the area under the ROC curve (AUC) and the area under the precision-recall (AUPR) curve [34]. The AUC and AUPR of a no-skill classifier were obtained from a random dataset based on the same distribution of the positive to negative classes in our study [35]. Spearman rank correlation coefficient analysis between candidate genera and immune factors was further used to assess their correlation.

### Statistical analyses

All statistical analyses were done and graphs created with R software (version 4.0.3). The Shapiro–Wilk test was used to determine the normality of continuous variables. Normally distributed data were described by means ± standard deviations. For inter-group comparisons at each timepoint, the Wilcoxon rank sum test was used to compare continuous variables, and Chi-square tests and Fisher's exact tests were used to analyze categorical variables. PERMANOVA was used to test for differences in beta diversity (measured by Bray–Curtis distance) between groups. For intra-group longitudinal comparisons of alpha diversity, a linear mixed effect model (LME) [36] was fitted for each alpha diversity index (Observed, Shannon, InvSimpson, and Chao1) in the good- and poor-response groups: lme (α-diversity index–timepoint, random =  ~ 1 + timepoint|patient ID, method = "ML"). Time-dependent effects were regarded as fixed effects, and both time-dependent effects and patient ID were included as random effects. Inter-rater agreement (kappa) was used for intra-group comparisons of categorical variables. The Benjamini–Hochberg method was used to correct for multiple testing. *P* values of < 0.05 were considered to indicate statistically significant differences.

## Results

### Cross-sectional and longitudinal variations of microbiome profiles in patients with good and poor response to nCCRT

**Fig. 1:**
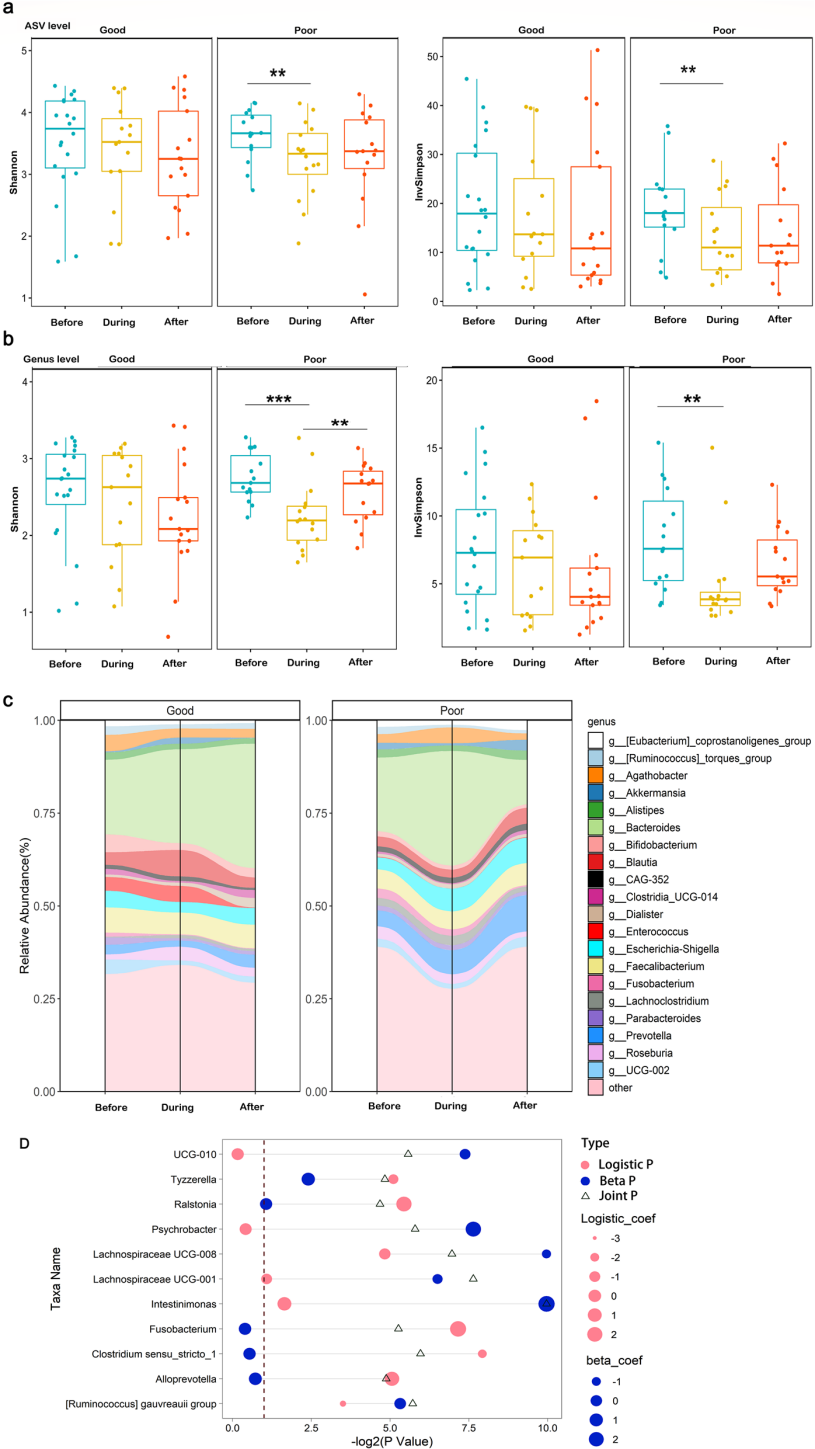
Changes in gut flora diversity and composition over the course of neoadjuvant concurrent chemoradiation therapy (nCCRT) for locally advanced rectal cancer. **a**, **b**, Bacterial α-diversity in the “good response” group (i.e., those with AJCC tumor regression grade [TRG] 0–1) and “poor response” group (AJCC TRG2-3) before, during, and after treatment, measured by the Shannon index (left) and the InvSimpson index (right) at the amplicon sequence variant (ASV) level (a) and the genus level (b). **c**, Changes in the abundance of the 20 most abundant genus-level microbes over time. *P < 0.05; **P < 0.01; ***P < 0.001

To determine whether and how nCCRT affects the gut microbiome, we obtained a total of 98 fecal samples from 39 patients with rectal cancer before, during, and after nCCRT. No significant differences were found at baseline (i.e., before nCCRT) for clinicopathologic factors (e.g., age, TNM disease stage, BMI, tumor length) between the good-response (TRG0-1) and poor-response (TRG2-3) groups (*P* > 0.05 for all, Fisher’s exact test, Table 1). After we successfully corrected for batch effects (Additional file 1: Fig. S1), our 16S rRNA gene sequencing analysis revealed that no clinicopathologic characteristic (e.g., age, sex, BMI) was associated with changes in the microbial profiles (Additional file 1: Fig. S2). Although no significant difference in alpha diversity was found between good and poor responders at any time relative to nCCRT (Figure S3), longitudinal analysis showed that the alpha diversity declined significantly, at both the amplicon sequence variant (ASV) and the genus levels, from before to during nCCRT among patients with poor response (Fig. 1a, b and Additional file 1: Fig. S4), whereas alpha diversity (assessed with the Shannon and InvSimpson indexes) remained stable throughout treatment among patients with good response (Fig. 1a, b). In addition, longitudinal changes in the top 20 most abundant genera were consistent with the changes in alpha diversity over time (Fig. 1c). Neither cross-sectional nor longitudinal analysis showed significant differences in beta diversity between the good- vs poor-response groups (Figure S5). Linear discriminant analysis effect size (LEfSe) identified 17 genera as differing between the good-response and poor-response groups (Figure S6), and zero-inflated beta regression with random effects (ZIBR) identified 11 genera as differing between the good- and poor-response groups (Additional file 2: Table S1). All of these results indicate that the microbiome profiles and changes in them from before to during nCCRT may correlate with outcome after nCCRT. To confirm whether receipt of induction chemotherapy before nCCRT affected the microbiome profiles, we analyzed taxa (by LEfSe analysis) and alpha and beta diversity. Thus even though differences in taxa were identified by LEfSe, the alpha and beta diversity analyses indicated that receipt of induction chemotherapy did not influence the microbiota profile compared with nCCRT (Additional file 1: Fig. S7).

**Table 1:** Clinicopathologic characteristics of the 39 enrolled patients

	TRG0-1 (n = 22)	TRG2-3 (n = 17)	*P* Value
Age, years, mean (sd)	55.9 (12.6)	61.1 (11.3)	0.184
Sex			0.318
Female	9	4	
Male	13	13	
Body mass index, mean (sd)	23.5 (3.00)	23.1 (2.67)	0.626
Family history of cancer			0.704
Yes	6	3	
No	16	14	
Tumor length, cm			0.194
≤ 5	12	13	
> 5	10	4	
Distance between anal verge and lower margin of tumor, cm			1
≤ 5	15	11	
> 5	7	6	
Colorectal polyps			0.738
Yes	7	7	
No	15	10	
CEA level, ng/mL			0.193
≤ 5	12	5	
> 5	10	12	
Induction chemotherapy*			0.334
Yes	10	11	
No	12	6	
Clinical T status			0.184
T3	6	9	
T4	16	8	
Clinical N status			0.054
N1	14	5	
N2	8	12	
Tumor differentiation			0.859
Low	3	2	
Medium	16	14	
High	3	1	
Vascular cancer thrombus			1
Yes	3	3	
No	19	14	
Nerve invasion			0.679
Yes	4	2	
No	18	15	

Classification variables are described statistically by composition, with *P* values determined by Fisher's exact test. The continuous variables age and body mass index were confirmed to be normally distributed, and so the results are expressed as mean (standard deviation), with *P* values determined by *t* tests

TRG, tumor regression grade (per the American Joint Committee on Cancer staging manual, 8th edn); CEA, carcinoembryonic antigen.

^*^Induction chemotherapy consisted of oral capecitabine 1000 mg/m^2^ twice daily for 14 days and intravenous oxaliplatin 130 mg/m^2^ on day 1

**Fig. 2:**
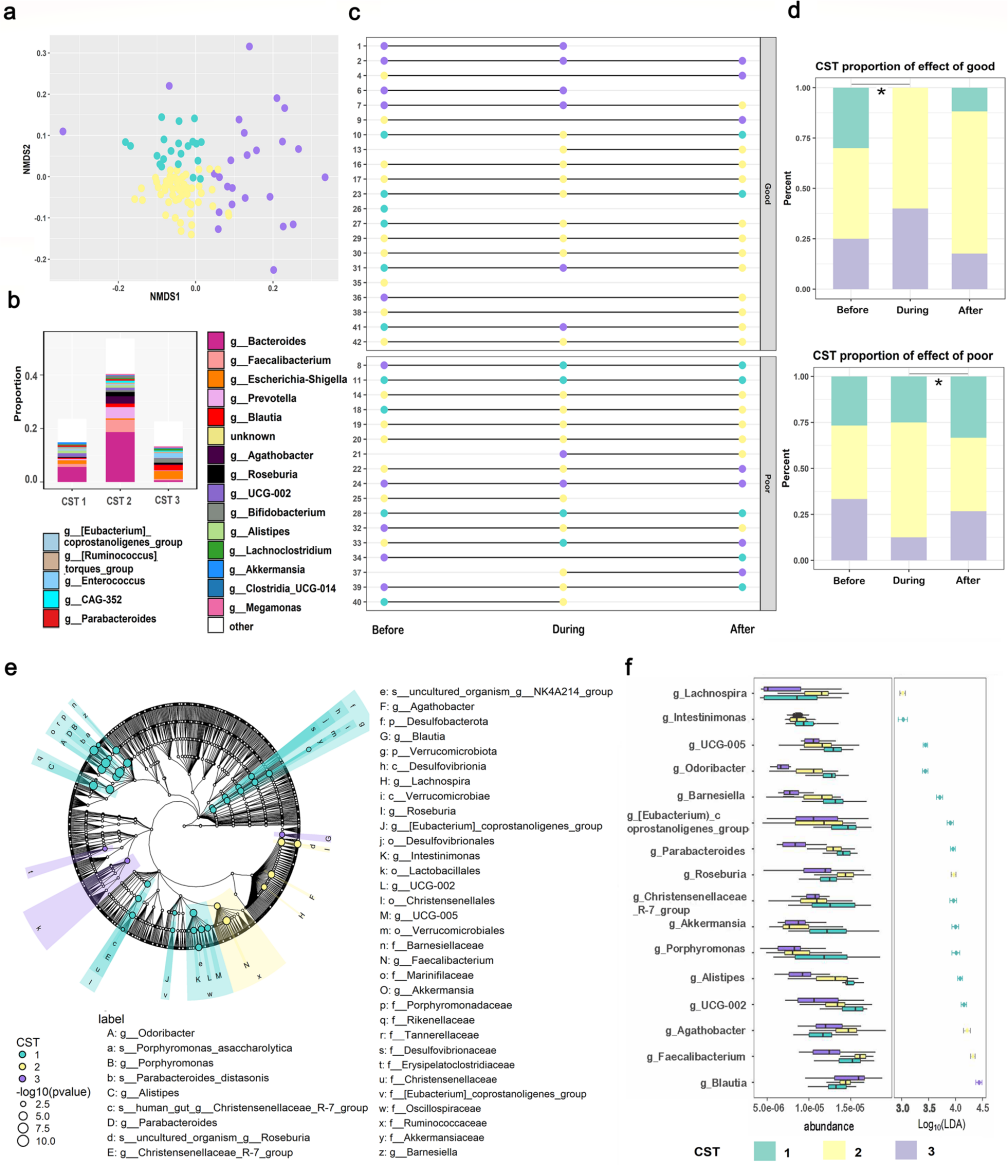
Longitudinal variations of enterotype in each patient with good or poor response to nCCRT. **a**, Nonmetric multidimensional scaling analysis of amplicon sequencing variants (ASVs) of all samples, indicating that all samples could be assigned to one of three community state types (CSTs). **b** Relative abundance of the top 19 most abundant genus-level microbes in the three CSTs. **c** Bacterial CSTs over time in individual patients before, during and after nCCRT. Patients were categorized in terms of response (good vs poor). **d** Overview of CST proportions in patients with good response to nCCRT (top) and poor response to nCCRT (bottom). *P < 0.05. No one in the good-response group displayed CST1 type during nCCRT. **e** Linear discriminant analysis effect size cladogram displaying the taxa enriched in CST1 (green), CST2 (yellow), and CST3 (purple). Taxa enriched within higher-level taxa are shown by darker shading. **f** Bacteria (at the genus level) showing the greatest changes in abundance in each CST

To further examine longitudinal variations in enterotype in each patient with good or poor response to nCCRT, we implemented an additional approach to identify distinct bacterial community patterns by using partitioning-around-medoids (PAM) clustering (see Methods). That analysis revealed three community state types (CSTs; denoted as purple, green, and yellow in Fig. 2a). The proportions of the top 19 abundant genera in each CST are shown in Fig. 2b. Longitudinal analysis indicated that the CSTs changed over time (Fig. 2c). Among the good responders, the CSTs were altered from before to during nCCRT (*P* = 0.036) but remained stable after nCCRT. However, among poor responders, CSTs changed significantly from during nCCRT to after nCCRT (*P* = 0.023). At the during-nCCRT measurement time, none of the good responders showed the CST1 enterotype (Fig. 2d). LEfSe analysis indicated that 11 genera (e.g., *Intestininonas*, *UCG-005, Akkermansia*, and others) were significantly enriched in CST1 (Fig. 2e, f). These findings suggest potential correlations between longitudinal changes in CSTs and effectiveness of therapy.

### Microbiome signature clusters based on host immune cells and immunomodulatory proteins correlated with outcomes after nCCRT

**Fig. 3:**
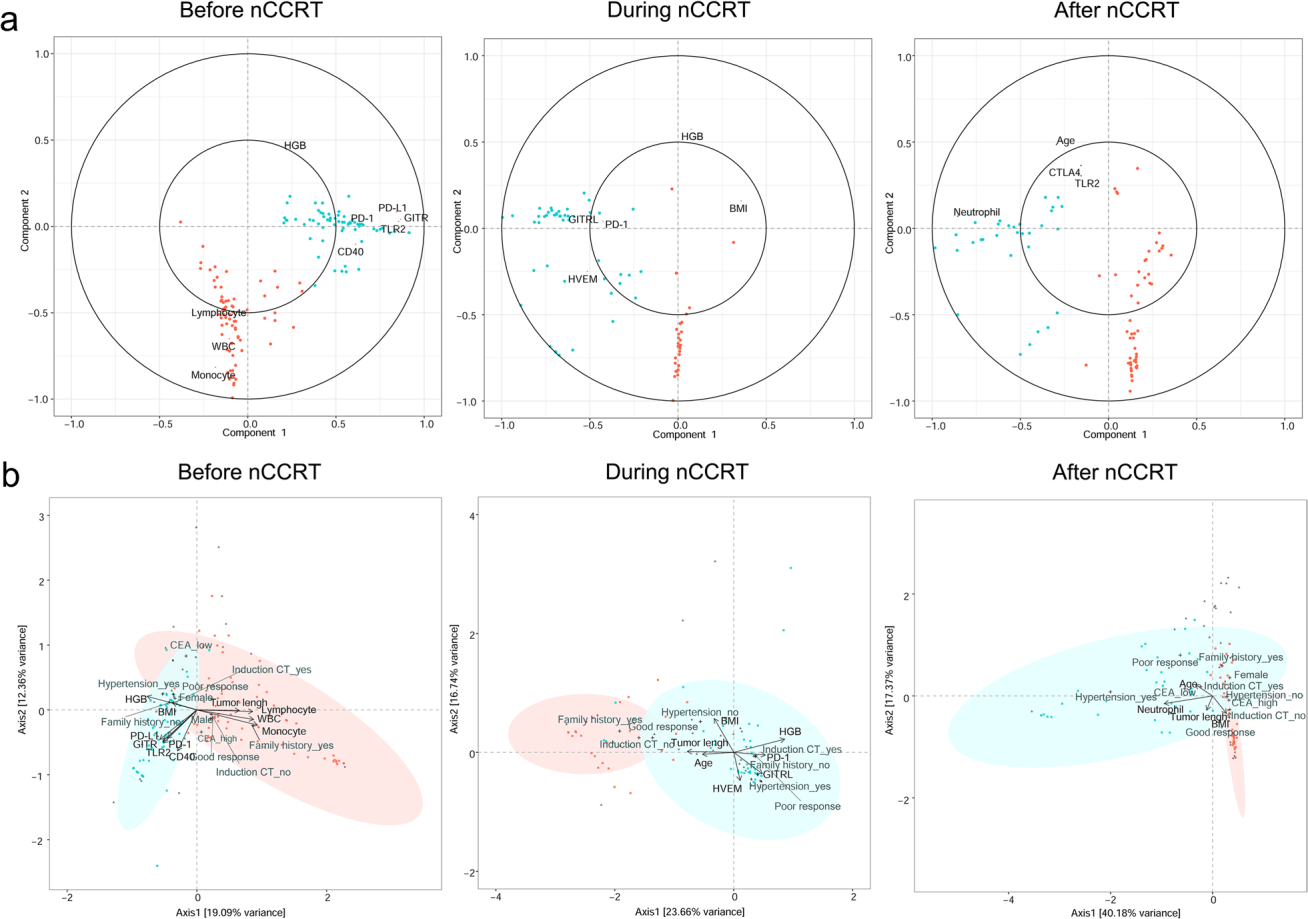
Sparse partial least squares (sPLS) regression and canonical correspondence analysis (CCpnA) of immune factors and intestinal bacterial taxa in patients undergoing neoadjuvant concurrent chemoradiation therapy (nCCRT). **a** Correlation circle plots for the first two sPLS dimensions, with correlations displayed for > 0.2– ≤  − 0.2. The two grey circles indicate correlation coefficient radii at 0.5 and 1.0. Bacterial amplicon sequence variants (ASVs) are displayed as dots, with color indicating subcluster affiliation (red, subcluster1; blue, subcluster2). Variables projected closely to each other are positively correlated. Variables projected diametrically opposite from each other are negatively correlated. Variables situated perpendicularly to each other are not correlated. **b** CCpnA of immune factors and intestinal bacterial taxa in patients undergoing nCCRT. Triplots showing dimensions 1 and 2 of the analysis that included continuous clinical variables (arrows), categorical variables ( +), and ASVs (circles). Samples are depicted as triangles. ASVs with a correlation of > 0.2– ≤ − 0.2 in the sPLS analysis were included in the canonical model. Only the variables and ASVs with a score > 0.2– ≤ − 0.2 in at least one canonical dimension are shown. The ASVs in the canonical plot are colored according to the subcluster they were affiliated with in the sPLS-based hierarchical clustering analysis

To gain insight into how immune factors (immune cells and immunomodulatory proteins) and gut microbial communities may interact with response to treatment, we used sparse partial least squares regression (sPLS) analysis and found two separate clusters at each timepoint (Fig. 3a). Loading plots of ASVs that contributed most to the subcluster1 and the subcluster2 at each timepoint are shown in Figure S8. We then used canonical correspondence analysis (CCpnA) to assess possible bidirectional associations between patient characteristics, immune cells, immunomodulatory proteins, and microbiota (Fig. 3b). Two clusters at each timepoint were found in CCpnA, and they were consistent with those in the sPLS regression. We observed that subcluster1 (red) was associated with good response to nCCRT, and subcluster2 (blue) was associated with poor response (Fig. 3b). At the before-nCCRT (baseline) measurement time, high numbers of white blood cells, lymphocytes, and monocytes tended to contribute to subcluster1, and high levels of PD-1, PD-L1, CD40, GITR, and TLR2 in peripheral blood tended to contribute to subcluster2 (which was also associated with poor response to therapy). During nCCRT, patients in subcluster2 had higher levels of PD-1, GITR, and the herpes virus entry mediator (HVEM/CD270). After nCCRT, however, patients in subcluster2 had higher neutrophil counts. Clustering the immune cells and immunomodulatory proteins by Spearman’s rank index for all patients revealed that the immune panel changed over time relative to nCCRT (Additional file 1: Fig. S9).

### Integrated prediction model combining microbial and immunological signatures stratified patients by clinical response to nCCRT

**Fig. 4:**
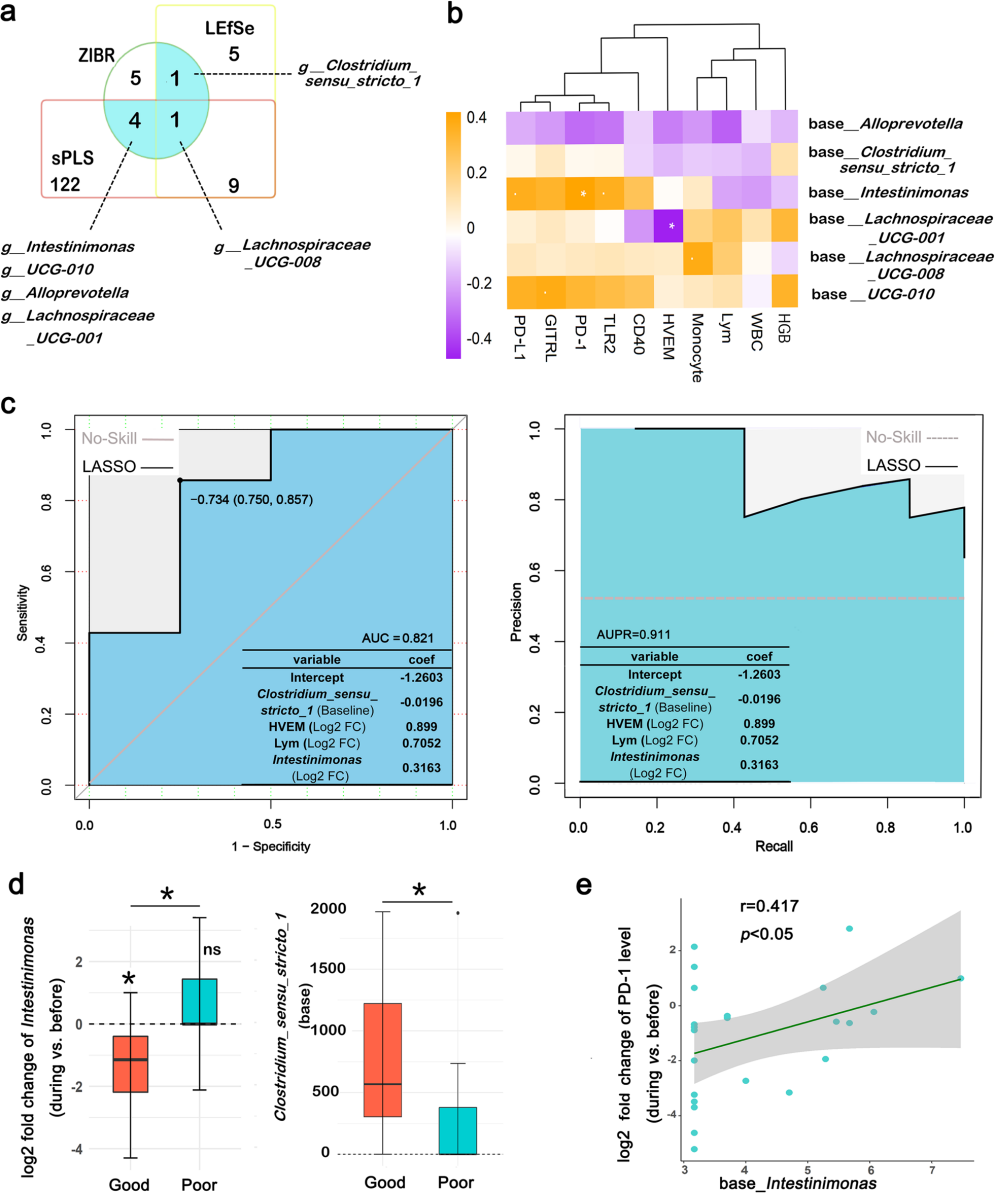
Integrated prediction model combining microbial and immunological signatures stratified according to response to nCCRT. **a** Venn plot of candidate microbes for prediction model construction. Candidate microbes were derived from 16 genera that differed between the good- and poor-response groups identified in cross-sectional analysis (LEfSe analysis before and during nCCRT), 11 different genera sourced from longitudinal analysis (ZIBR analysis), and 136 genera that contributed to microbial clustering in multivariate statistical analysis (sPLS before and during nCCRT). Finally, 6 genera (cyan background) were used for model construction. **b** Hierarchical clustering plot of correlation between baseline levels of candidate genera and log2-transformed fold-change of immune cells and immunomodulatory proteins from before to during nCCRT. Color scale represents the Spearman rank correlation coefficient. **c** Areas under the receiver operating characteristic (ROC) curve (left) and the precision-recall curve (right) to measure model performance in the validation set, with variables and coefficients as shown. **d** Evaluation of differences in log2-transformed fold-change of *Intestinimonas* from before nCCRT to during nCCRT and the baseline level of *Clostridium *sensu stricto* 1* between the good- and poor-response groups. e. Scatter plot of baseline level of *Intestinimonas* showed positive association with log2-transformed fold-change in PD-1 levels from before to during nCCRT. *P < 0.05; **P < 0.01; ***P < 0.001

To further investigate the relationship between bacterial diversity, immune panel, and treatment response, candidate microbes (screened by sPLS, LEfSe, and ZIBR) and immune cells and immunomodulatory proteins (screened by Adonis) were selected to construct a predictive LASSO model at the before-nCCRT and during-nCCRT timepoints. However, the prediction models classified by specific nCCRT timepoint did not have sufficient power to predict TRG (data not shown), suggesting that signatures at a single measurement time would not directly reflect the final effectiveness of nCCRT. Taking this into account, we included the following variables, measured at two timepoints (before and during nCCRT): baseline levels and log2-transformed fold-changes in 6 genera (Fig. 4a, cyan), 3 immune cells, 7 immunomodulatory proteins, and hemoglobin) in the LASSO model. We then analyzed potential correlations between baseline levels of the candidate genera and log2-transformed fold changes in the immune cells and immunomodulatory proteins comparing before-nCCRT with during-nCCRT (Fig. 4b). We found that an integrated model containing four signatures (Fig. 4c), including *Clostridium *sensu stricto* 1* abundance at baseline levels, log2-transformed fold-change in levels of HVEM/CD270, lymphocyte counts, and *Intestinimonas* abundance, could accurately predict outcomes after nCCRT in the validation set (area under the precision-recall curve [AUPC] = 0.911, area under the receiver operating characteristic curve [AUC] = 0.821, Fig. 4c). In this model (Fig. 4c), low baseline abundance of *Clostridium *sensu stricto* 1*, increased fold change of HVEM levels, and lymphocyte counts as well as decreased fold change in *Intestinimonas* abundance before nCCRT vs during nCCRT contributed to poor response. Moreover, *Intestinimonas* during nCCRT was decreased relative to before nCCRT in the good response group, and its dynamic change was distinct between good- and poor-response groups (Fig. 4d). The relative abundance of *Clostridium *sensu stricto* 1* was higher in good response group compared with the poor response group before nCCRT (Fig. 4d). Correlation plots of candidate genera, immune cells, and immunomodulatory proteins showed that baseline level of *Lachnospiraceae UCG-001* was negatively associated with log2-transformed fold change of HVEM levels from before to during nCCRT, whereas baseline level of *Intestinimonas* was positively associated with log2-transformed fold change in PD-1 levels from before to during nCCRT (Fig. 4b, e).

## Discussion

Active interactions of the microbiota with chemoradiotherapy and immunotherapy have been well recognized to affect the complex interactions between the microbiome and host immune response [37], [7, 12]. Prospective prediction of the effectiveness of nCCRT, the standard therapy for LARC, requires investigations of the dynamic variations in microbiome and immune factors, identification of key signatures and their biological relevance, and clarification of their multivariate correlations with treatment outcome. Our cross-sectional and longitudinal analyses of gut microbiome and immunomodulators in fecal and blood samples from patients with LARC revealed dynamic microbiome variations and characteristic enterotypes in the good-response and poor-response groups. Microbiome signatures clustered based on host immune cells and immunomodulatory proteins correlated with outcomes after nCCRT. Our integrated predictive model combining microbial and immunological signatures could efficiently stratify patients in terms of response to nCCRT.

Both chemotherapy and radiotherapy can influence the diversity and abundance of intestinal microbiota [6, 38, 39]. Several platinum-based compounds can change the proportions of components of the microbiome [39, 40]. Cisplatin in particular has been shown to inhibit the growth of 29 microbes (7 Gram-negative and 8 Gram-positive bacterial strains, 7 yeast strains, and 7 mold strains) [41]. In a C57BL/6 mouse model, cisplatin caused gut microbiota dysbiosis according to 16S rRNA gene sequencing and metabolomic analysis [39]. Another platinum-based compound, carboplatin, was found to reduce the abundance of several taxa in patients with ovarian cancer [42]. Moreover, capecitabine and oxaliplatin (CAPEOX) was shown by 16S rRNA gene sequencing to have drastic effects on the intestinal microbiota [43]. In another study, both radical surgery and capecitabine + oxaliplatin had non-negligible effects on the gut microbiota in patients with colorectal cancer [43]. In our study, receipt of induction chemotherapy (a single cycle of capecitabine + oxaliplatin) did not affect the gut microbiota. Chi-square tests showed no significant differences in the proportions of receipt of induction chemotherapy between the two groups (Chi-square = 0.76, *P* > 0.05). It suggested that only one cycle of induction chemotherapy before nCCRT did not enhance short-term prognosis.

As for radiotherapy, we previously showed that the interaction between the gut microbiome and radiotherapy is bidirectional, in that radiotherapy can disrupt the microbiome and those disruptions can in turn influence the effectiveness of anticancer therapy [44]. Because concurrent radiotherapy and chemotherapy is the standard of care for LARC, it is difficult to explore the separate influences of chemotherapy and radiotherapy on the gut microbiota, but use of mouse models would be feasible to explore these different influences. Considering the potential batch effect from sequencing samples twice, we next used the ComBat method to correct for the batch effect [20, 22], which has been used for this purpose in previous studies. Previous studies have determined that removing batch effects could reduce dependence, stabilize error rate estimates, and improve reproducibility [21]. After having done so with the ComBat method, we re-ran the LEfSe, alpha and beta diversity analyses to determine whether the batch effect had been successfully removed. This batch effect correction did not affect the lack of difference in alpha diversity between two batches (Figure S1a); the Adonis results showed that the variance (R2) explained by the batch effect decreased from 0.050 to 0.043. This correction removed any difference in beta diversity between the two batches (*P* > 0.05) (Figure S1b) and reduced the number of taxa that differed between two batches (Figure S1c) which successfully removed any difference in beta diversity between the two batches (*P* = 0.667) and reduced the number of taxa that differed between two batches.

Intestinal microbiota are known to affect immune status and have been linked with outcomes after chemoradiation and immunotherapy [45, 46]. Our predictive model integrated baseline and early-in-treatment changes in microbial and immunological signatures and immunocytes during chemoradiation to allow prospective prediction of outcome after nCCRT. Among all of the bacterial signatures, *Intestininonas* and *Clostridium *sensu stricto* 1* deserve special attention for their notable contributions to our prediction model. Interestingly, baseline level of *Intestininonas* was positively associated with fold changes in PD-1 (r = 0.417, *P* = 0.031), an immune checkpoint protein used widely in cancer therapy. Our findings suggest that particular microbial species could interfere with immune system function and potentially be useful in combinations of chemoradiation and anti-PD-1 immunotherapy to enhance clinical outcomes in patients with LARC.

This study had several limitations deserving further analysis and discussion. First, although we obtained fecal and serum samples at several times relative to treatment, the number of enrolled patients (n = 39) was relatively small and not all patients provided samples at all timepoints. Larger numbers of patients and more complete data collection are needed to verify our results and improve our model. Second, we used only TRG as an endpoint after nCCRT, and long-term outcomes including recurrence and survival need to be further analyzed. Third, we used soluble immune markers in patients’ blood samples to identify the patients’ immunological status, and flow cytometry may be more effective for evaluating the expression of certain immune biomarkers in specific immune subsets. Fourth, the taxonomic scope of our data is limited; fungi, which have been implicated in the response to radiation treatment [47], were not included in the analysis. Finally, although we assessed correlations between immunomodulation indexes and genera, further experiments with animal models and cell cultures are warranted to determine causality and mechanisms.

## Conclusions

A deeper understanding of the effects of nCCRT on the gut microbiota, and vice versa, is still needed. We found that nCCRT affected the diversity, abundance, and composition of the gut microbiome in patients with LARC. Fecal microbiome diversity decreased throughout the treatment period, and those patients with a good response to that treatment had higher richness at the integral level. Most patients had shifts in dominant bacteria over the entire treatment, but some remained relatively stable. We conclude that nCCRT does affect the fecal microbiome, and that the fecal microbiome of LARC patients differs according to sensitivity (or resistance) to nCCRT.

## Supplementary Information

**Additional file 1: Figure S1. **Successful correction of the batch effect. The ComBat method was used for batch effect correction in microbiome data; the alpha diversity (a), beta diversity (b), and LEfSe (c) analyses were conducted before (left) and after (right) batch effect removal. **Figure S2. **The beta diversity of gut microbiomes for patients categorized by clinicopathologic characteristics (e.g., age, sex, body mass index [BMI]); all *P *values were >0.05. **Figure S3**. Cross-sectional analysis of alpha diversity in good- and poor-response groups before, during, and after neoadjuvant concurrent chemoradiation therapy (nCCRT). **Figure S4. **Alpha diversity measured by the Chao1 index (left panels) and Observed index (right panels) of gut bacteria before, during, and after neoadjuvant concurrent chemoradiation therapy (nCCRT). **Figure S5. **Longitudinal and cross-sectional analysis of gut microbiome β-diversity. **a**, Longitudinal analysis of gut microbiome β-diversity in the good- and poor-response groups. **b-d**, Cross-sectional analysis of gut microbiome β-diversity in the good- and poor-response groups before (**b**), during (**c**), and after (**d**) neoadjuvant concurrent chemoradiation therapy (nCCRT). **e**, Gut microbiome β-diversity at all three measurement points. **Figure S6. **Differences in the abundance of taxa in the gut microbiota between the good-response group (blue) and the poor-response group (red) before, during, and after neoadjuvant concurrent chemoradiation therapy (nCCRT). **Figure S7**. Receipt of induction chemotherapy did not affect the gut microbiome profile, as indicated by (**a**) alpha diversity, (b) beta diversity, and (c) LEfSe analyses. **Figure S8**. Loading plots of amplicon sequence variants (ASVs) with the greatest contributions to the subcluster1 (left, red) and subcluster2 (right, blue) before, during, and after neoadjuvant concurrent chemoradiation therapy (nCCRT). **Figure S9. **Correlations between immune cells and immunomodulatory proteins before, during, and after neoadjuvant concurrent chemoradiation therapy (nCCRT). Spearman correlation coefficients were used to describe correlations between each pair of immune factors. Positive and negative correlations are represented by red (positive) and blue (negative) circles, and the size of the circles and their color intensity indicate the strength of the correlation. *P<0.05.

Additional file 2: **Table S1**. Taxa identified by ZIBR as differing between the good- and poor-response groups. **Table S2.** Numbers of samples collected from patients before, during, and after neoadjuvant concurrent chemoradiation therapy.

## Data Availability

The 16S rRNA gene sequences are available through Bio-Med Big Data Center (BMDC) under project IDEP002914. The datasets are available at https://www.biosino.org/node/project/detail/OEP002914

## Abbreviations

AJCC: American joint committee on cancer
ASVs: Amplicon sequence variants
AUC: Area under the [receiver-operating characteristics] curve
AUPR: Area under the precision-recall curve
BMI: Body mass index
CCpnA: Canonical correspondence analysis
CSTs: [Microbial] community state types
HVEM/CD270: Herpes virus entry mediator
IL: Interleukin
LARC: Locally advanced rectal cancer
LASSO: Least absolute shrinkage and selection operator
LEfSe: Linear discriminant analysis effect size
nCCRT: Neoadjuvant concurrent chemoradiotherapy
OTU: Operational taxonomic unit
PAM: Partitioning-around-medoid
pCR: Pathologic complete response
PERMANOVA: Permutational analysis of variance
ROC: Receiver-operating characteristic
sPLS: Sparse partial least squares regression
TRG: Tumor regression grade
ZIBR: Zero-inflated beta regression with random effects
