# Supplementary material for: Prospective, longitudinal analysis of the gut microbiome in patients with locally advanced rectal cancer predicts response to neoadjuvant concurrent chemoradiotherapy
Source: J Transl Med. 2023 Mar 26;21:221. doi: 10.1186/s12967-023-04054-1 (PMC10041716; doi:10.1186/s12967-023-04054-1)
Supplement: Supplementary file 1 — Additional file 1: Figure S1. Successful correction of the batch effect. The ComBat method was used for batch effect correction in microbiome data; the alpha diversity (a), beta diversity (b), and LEfSe (c) analyses were conducted before (left) and after (right) batch effect removal. Figure S2. The beta diversity of gut microbiomes for patients categorized by clinicopathologic characteristics (e.g., age, sex, body mass index [BMI]); all P values were >0.05. Figure S3. Cross-sectional analysis of alpha diversity in good- and poor-response groups before, during, and after neoadjuvant concurrent chemoradiation therapy (nCCRT). Figure S4. Alpha diversity measured by the Chao1 index (left panels) and Observed index (right panels) of gut bacteria before, during, and after neoadjuvant concurrent chemoradiation therapy (nCCRT). Figure S5. Longitudinal and cross-sectional analysis of gut microbiome β-diversity. a, Longitudinal analysis of gut microbiome β-diversity in the good- and poor-response groups. b-d, Cross-sectional analysis of gut microbiome β-diversity in the good- and poor-response groups before (b), during (c), and after (d) neoadjuvant concurrent chemoradiation therapy (nCCRT). e, Gut microbiome β-diversity at all three measurement points. Figure S6. Differences in the abundance of taxa in the gut microbiota between the good-response group (blue) and the poor-response group (red) before, during, and after neoadjuvant concurrent chemoradiation therapy (nCCRT). Figure S7. Receipt of induction chemotherapy did not affect the gut microbiome profile, as indicated by (a) alpha diversity, (b) beta diversity, and (c) LEfSe analyses. Figure S8. Loading plots of amplicon sequence variants (ASVs) with the greatest contributions to the subcluster1 (left, red) and subcluster2 (right, blue) before, during, and after neoadjuvant concurrent chemoradiation therapy (nCCRT). Figure S9. Correlations between immune cells and immunomodulatory proteins before, during, and after neoadj [file 12967_2023_4054_MOESM1_ESM.pdf]

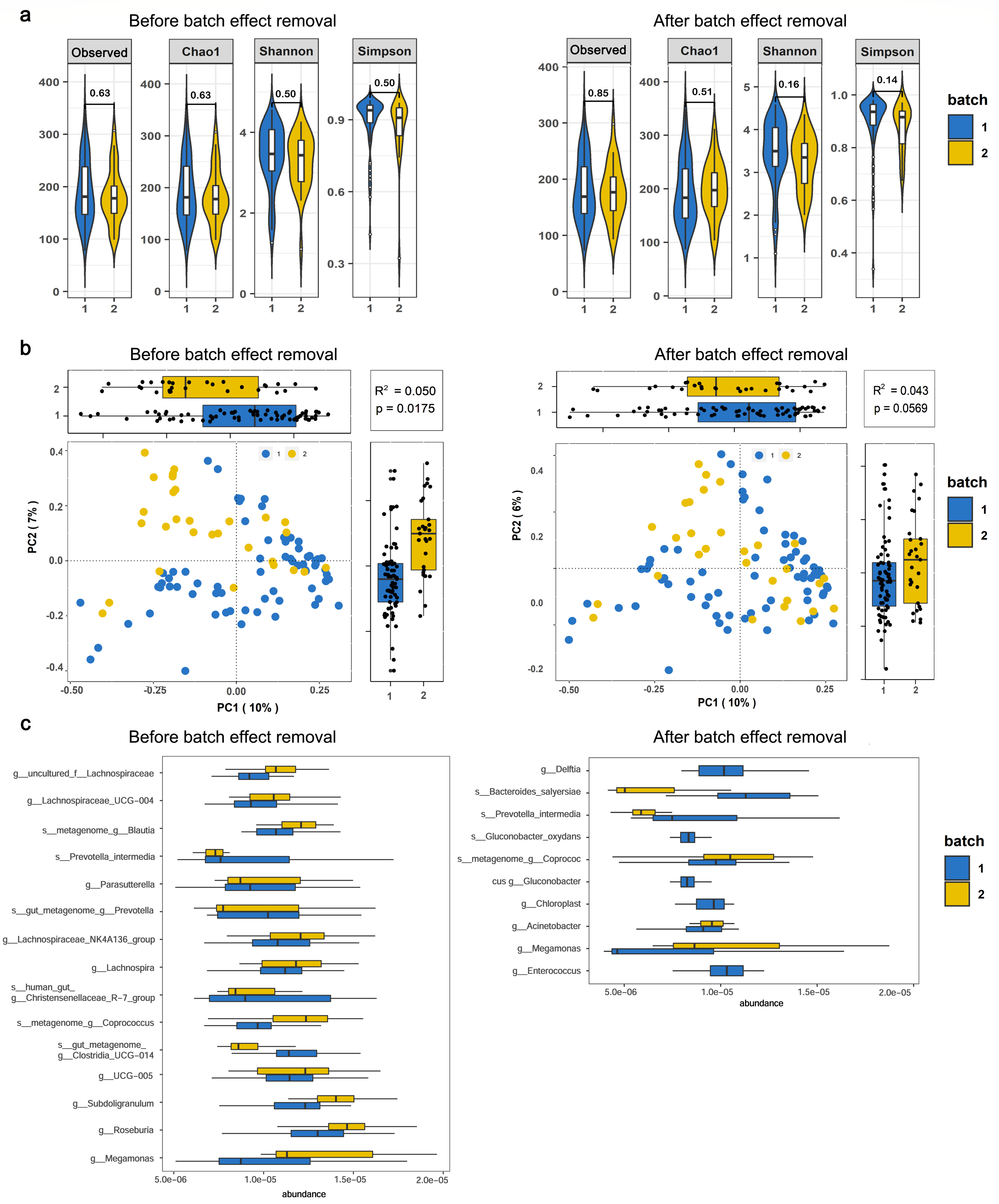
a
Before batch effect removal
Observed
Chao1
Shannon
Simpson
0.63
0.63
0.50
0.50
After batch effect removal
Observed
Chao1
Shannon
Simpson
0.85
0.51
0.16
0.14
batch
1
2
b
Before batch effect removal
R² = 0.050
p = 0.0175
PC2 (7%)
PC1 (10%)
After batch effect removal
R² = 0.043
p = 0.0569
PC2 (6%)
PC1 (10%)
batch
1
2
c
Before batch effect removal
g__uncultured_f__Lachnospiraceae
g__Lachnospiraceae_UCG-004
s__metagenome_g__Blautia
s__Prevotella_intermedia
g__Parasutterella
s__gut_metagenome_g__Prevotella
g__Lachnospiraceae_NK4A136_group
g__Lachnospira
s__human_gut_
g__Christensenellaceae_R-7_group
s__metagenome_g__Coprococcus
s__gut_metagenome_
g__Clostridia_UCG-014
g__UCG-005
g__Subdoligranulum
g__Roseburia
g__Megamonas
5.0e-06
1.0e-05
1.5e-05
2.0e-05
abundance
After batch effect removal
g__Delftia
s__Bacteroides_salyersiae
s__Prevotella_intermedia
s__Gluconobacter_oxydans
s__metagenome_g__Coprococ
cus g__Gluconobacter
g__Chloroplast
g__Acinetobacter
g__Megamonas
g__Enterococcus
5.0e-06
1.0e-05
1.5e-05
2.0e-05
abundance
batch
1
2

FigureS1

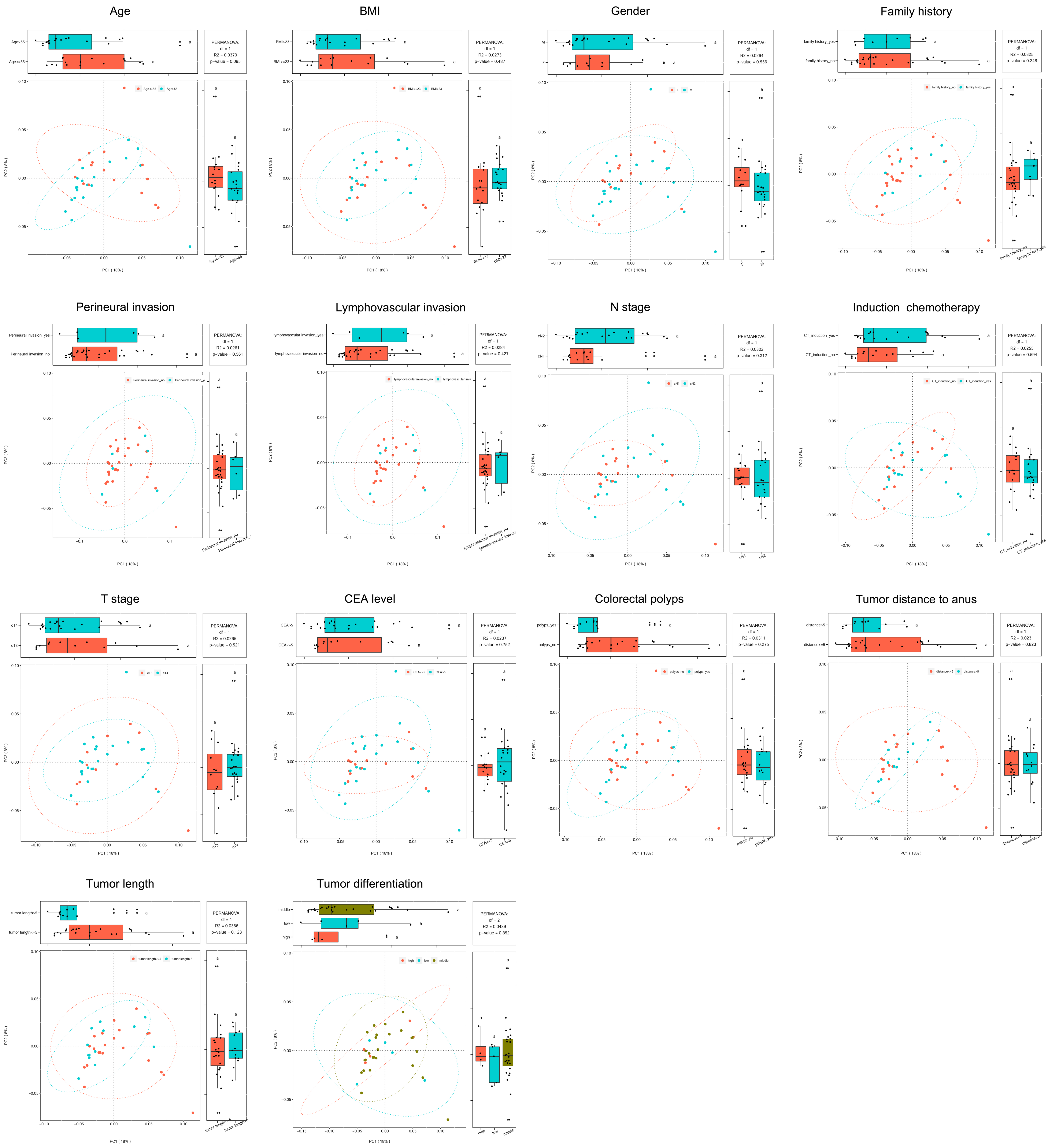
Age
PERMANOVA: df = 1 R2 = 0.0379 p−value = 0.085
BMI
PERMANOVA: df = 1 R2 = 0.0273 p−value = 0.487
Gender
PERMANOVA: df = 1 R2 = 0.0264 p−value = 0.556
Family history
PERMANOVA: df = 1 R2 = 0.0325 p−value = 0.248
Perineural invasion
PERMANOVA: df = 1 R2 = 0.0261 p−value = 0.561
Lymphovascular invasion
PERMANOVA: df = 1 R2 = 0.0284 p−value = 0.427
N stage
PERMANOVA: df = 1 R2 = 0.0302 p−value = 0.312
Induction chemotherapy
PERMANOVA: df = 1 R2 = 0.0255 p−value = 0.594
T stage
PERMANOVA: df = 1 R2 = 0.0265 p−value = 0.521
CEA level
PERMANOVA: df = 1 R2 = 0.0237 p−value = 0.752
Colorectal polyps
PERMANOVA: df = 1 R2 = 0.0311 p−value = 0.275
Tumor distance to anus
PERMANOVA: df = 1 R2 = 0.023 p−value = 0.823
Tumor length
PERMANOVA: df = 1 R2 = 0.0366 p−value = 0.123
Tumor differentiation
PERMANOVA: df = 2 R2 = 0.0439 p−value = 0.852
PC1 (18%)
PC2 (8%)

Figure S2

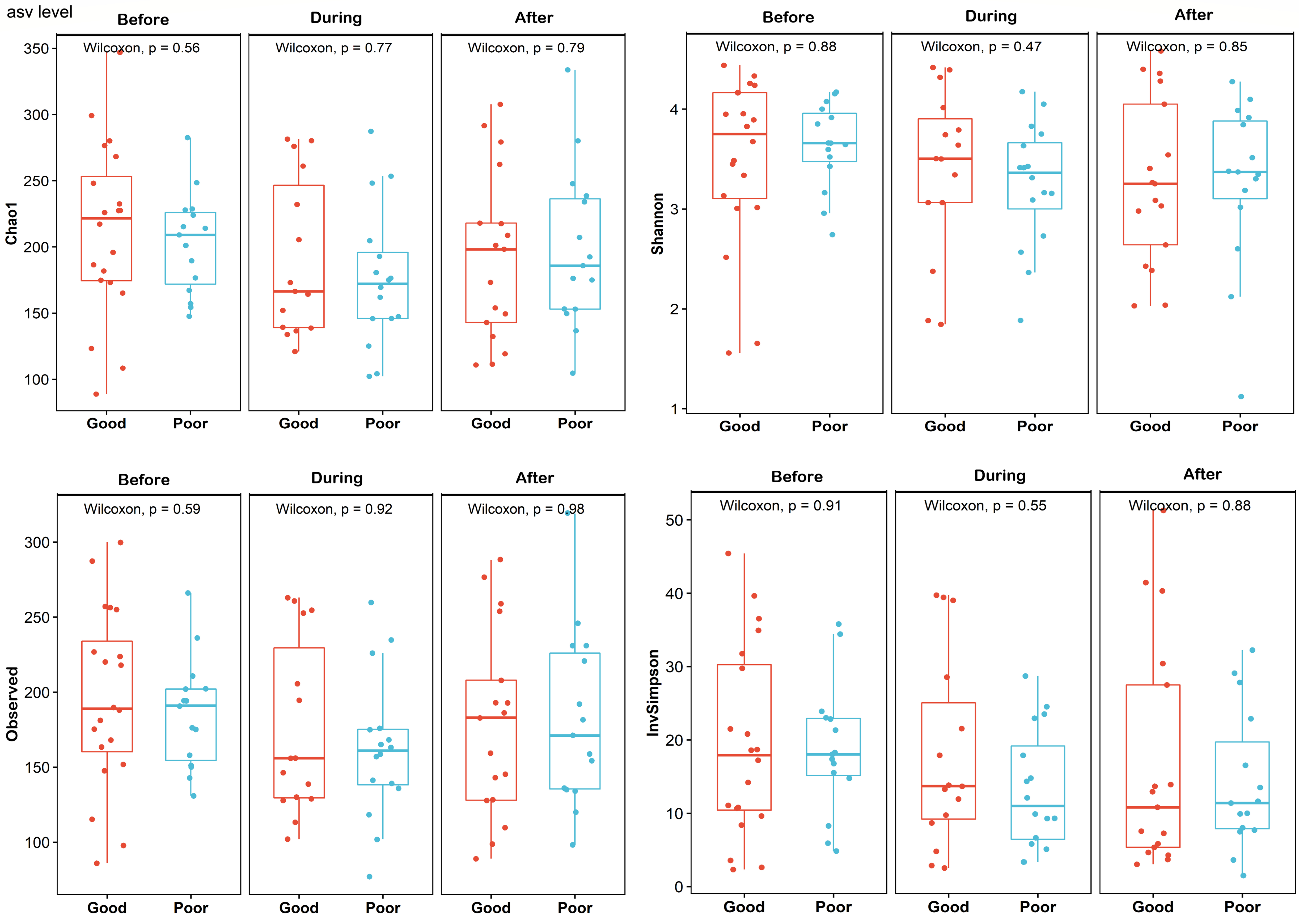
asv level
Before
During
After
Wilcoxon, p = 0.56
Wilcoxon, p = 0.77
Wilcoxon, p = 0.79
Chao1
Good
Poor
Before
During
After
Wilcoxon, p = 0.88
Wilcoxon, p = 0.47
Wilcoxon, p = 0.85
Shannon
Good
Poor
Before
During
After
Wilcoxon, p = 0.59
Wilcoxon, p = 0.92
Wilcoxon, p = 0.98
Observed
Good
Poor
Before
During
After
Wilcoxon, p = 0.91
Wilcoxon, p = 0.55
Wilcoxon, p = 0.88
InvSimpson
Good
Poor

FigureS3

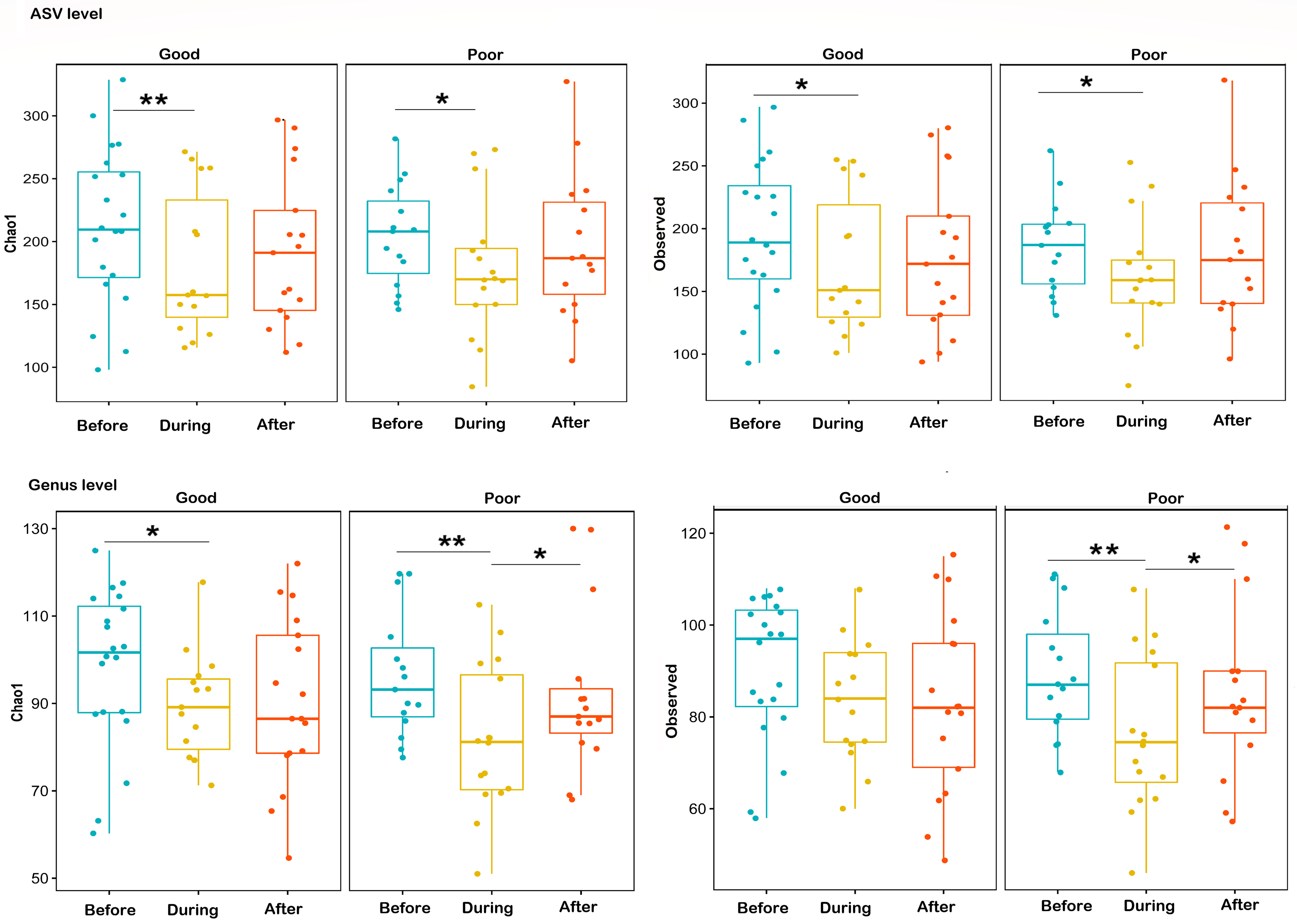

ASV level
Good
Poor
Chao1
Observed
Before
During
After
**
*
Genus level

FigureS4

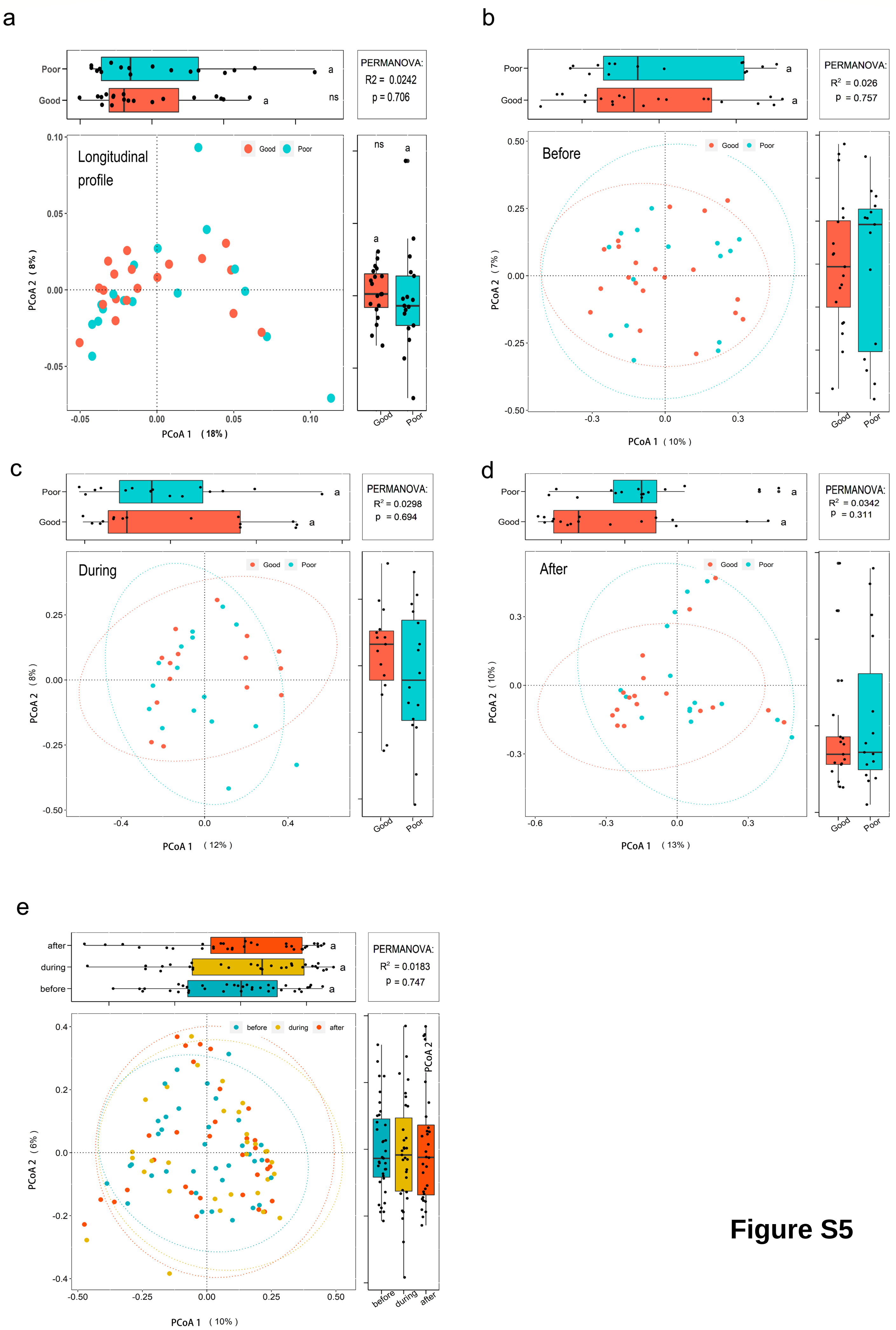
a
Poor
Good
PERMANOVA:
R2 = 0.0242
p = 0.706
ns
Longitudinal profile
Good
Poor
PCoA 2 (8%)
PCoA 1 (18%)
b
PERMANOVA:
R^2 = 0.026
p = 0.757
Before
PCoA 2 (7%)
PCoA 1 (10%)
c
PERMANOVA:
R^2 = 0.0298
p = 0.694
During
PCoA 2 (8%)
PCoA 1 (12%)
d
PERMANOVA:
R^2 = 0.0342
p = 0.311
After
PCoA 2 (10%)
PCoA 1 (13%)
e
after
during
before
PERMANOVA:
R^2 = 0.0183
p = 0.747
PCoA 2 (6%)
PCoA 1 (10%)

Figure S5

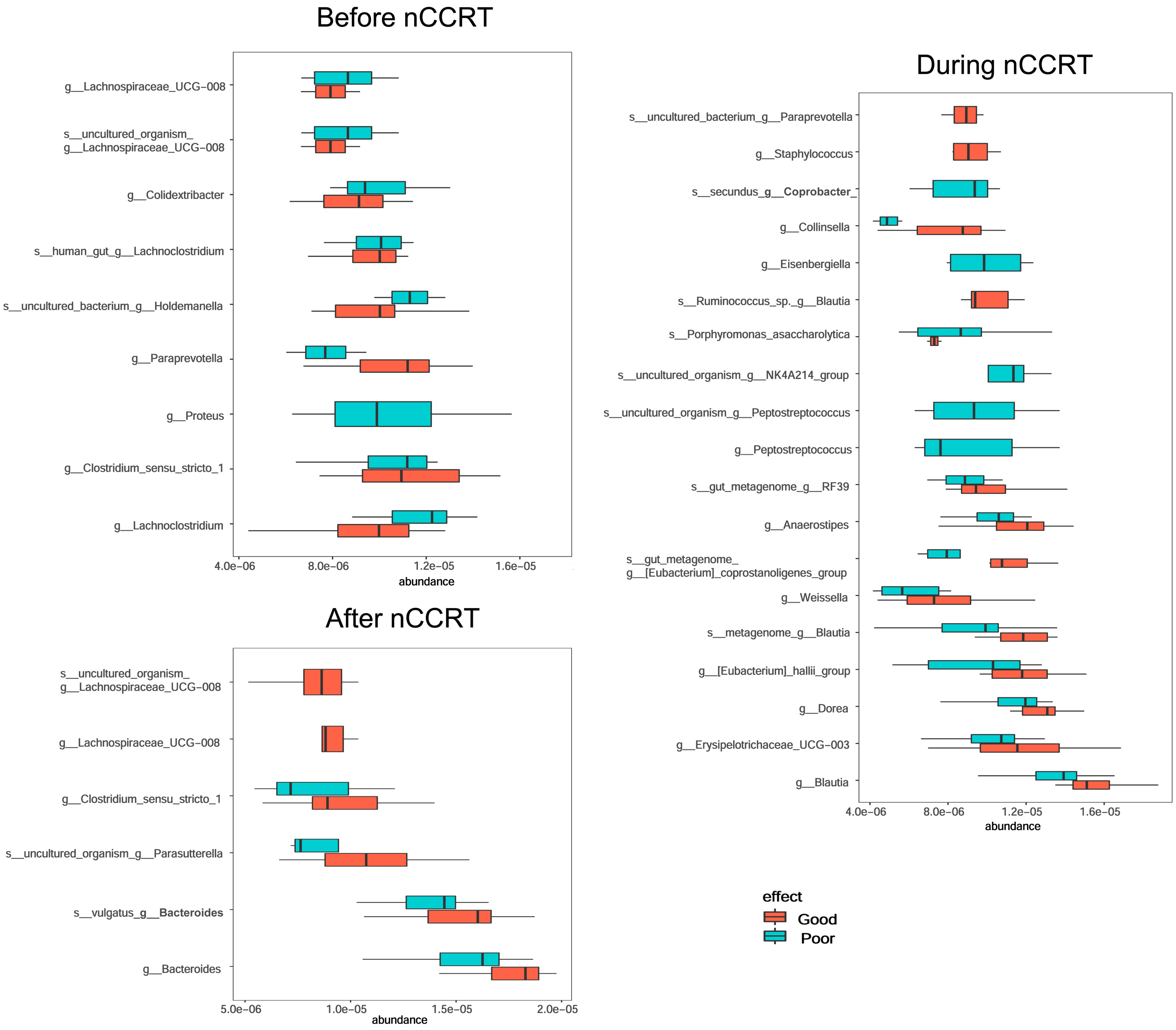
Before nCCRT
g__Lachnospiraceae_UCG−008
s__uncultured_organism_
g__Lachnospiraceae_UCG−008
g__Colidextribacter
s__human_gut_g__Lachnoclostridium
s__uncultured_bacterium_g__Holdemanella
g__Paraprevotella
g__Proteus
g__Clostridium_sensu_stricto_1
g__Lachnoclostridium
4.0e−06
8.0e−06
1.2e−05
1.6e−05
abundance
After nCCRT
s__uncultured_organism_
g__Lachnospiraceae_UCG−008
g__Lachnospiraceae_UCG−008
g__Clostridium_sensu_stricto_1
s__uncultured_organism_g__Parasutterella
s__vulgatus_g__Bacteroides
g__Bacteroides
5.0e−06
1.0e−05
1.5e−05
2.0e−05
abundance
During nCCRT
s__uncultured_bacterium_g__Paraprevotella
g__Staphylococcus
s__secundus_g__Coprobacter
g__Collinsella
g__Eisenbergiella
s__Ruminococcus_sp._g__Blautia
s__Porphyromonas_asaccharolytica
s__uncultured_organism_g__NK4A214_group
s__uncultured_organism_g__Peptostreptococcus
g__Peptostreptococcus
s__gut_metagenome_g__RF39
g__Anaerostipes
s__gut_metagenome_
g__[Eubacterium]_coprostanoligenes_group
g__Weissella
s__metagenome_g__Blautia
g__[Eubacterium]_hallii_group
g__Dorea
g__Erysipelotrichaceae_UCG−003
g__Blautia
4.0e−06
8.0e−06
1.2e−05
1.6e−05
abundance
effect
Good
Poor

FigureS6

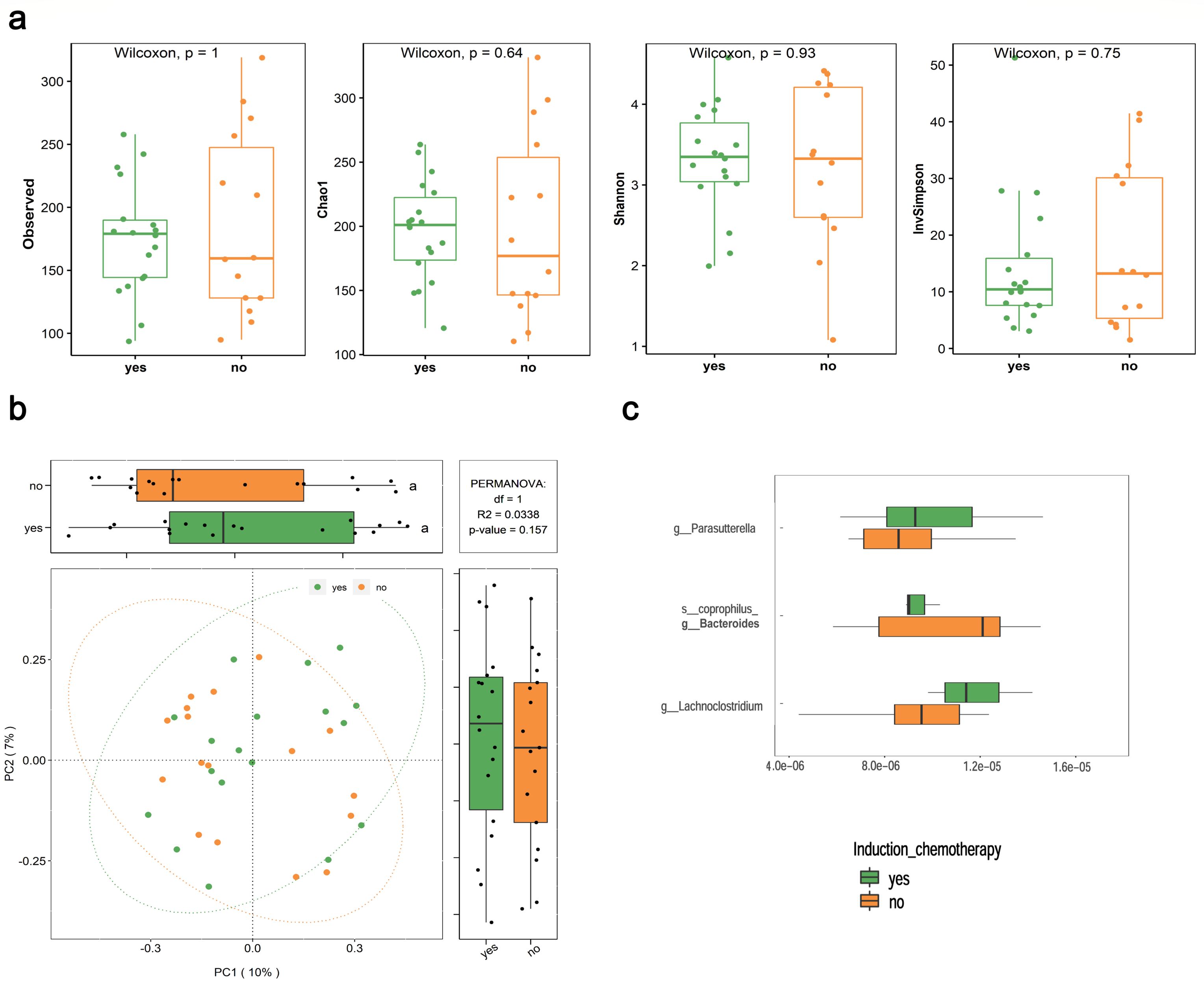
a
Wilcoxon, p = 1
Observed
300
250
200
150
100
yes
no
Wilcoxon, p = 0.64
Chao1
300
250
200
150
100
yes
no
Wilcoxon, p = 0.93
Shannon
4
3
2
1
yes
no
Wilcoxon, p = 0.75
InvSimpson
50
40
30
20
10
0
yes
no
b
no
yes
a
a
PERMANOVA:
df = 1
R2 = 0.0338
p-value = 0.157
yes
no
PC2 (7%)
0.25
0.00
-0.25
-0.3
0.0
0.3
PC1 (10%)
yes
no
c
g__Parasutterella
s__coprophilus_
g__Bacteroides
g__Lachnoclostridium
4.0e−06
8.0e−06
1.2e−05
1.6e−05
Induction_chemotherapy
yes
no

FigureS7

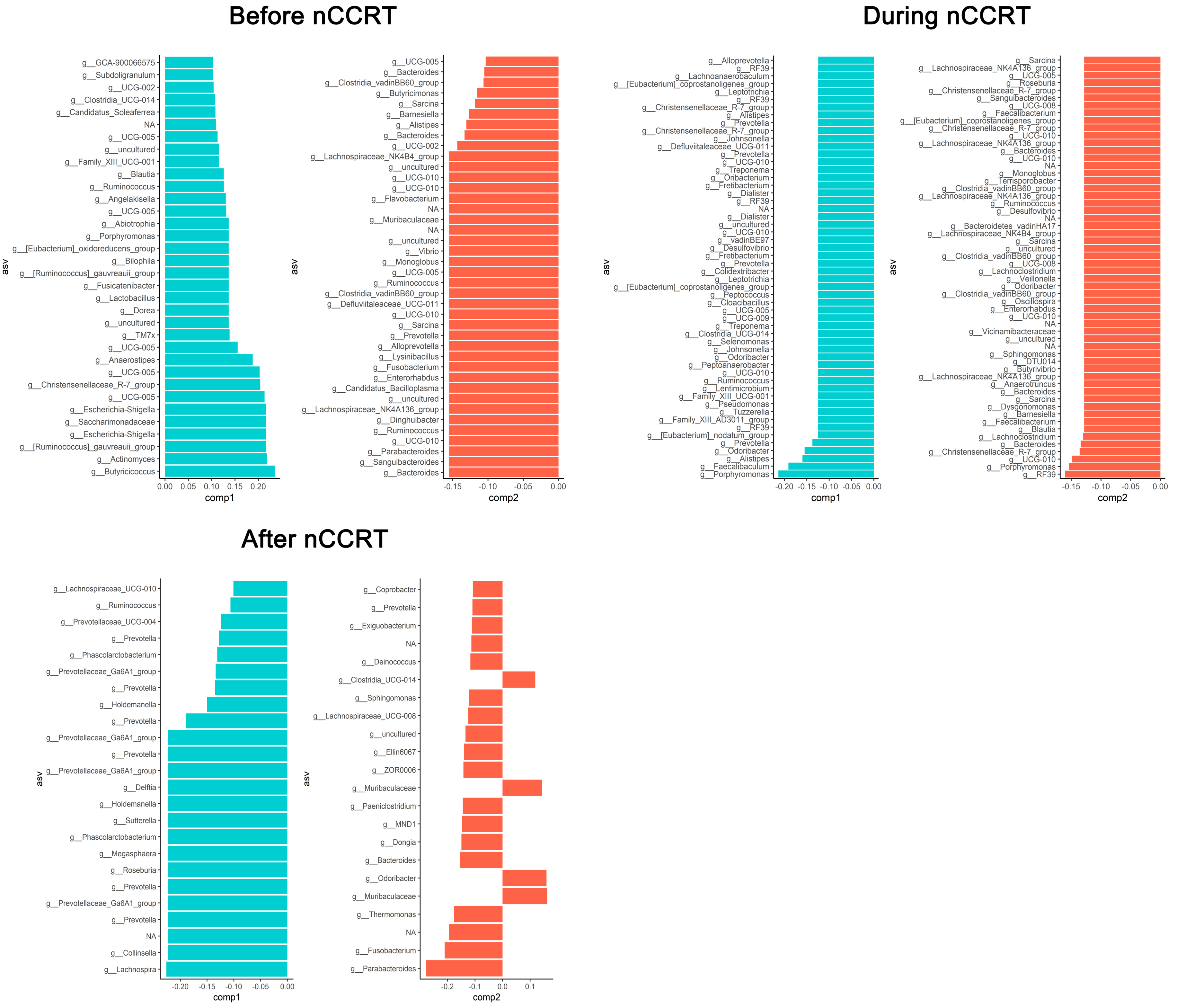
Before nCCRT
g__GCA-900066575
g__Subdoligranulum
g__UCG-002
g__Clostridia_UCG-014
g__Candidatus_Soleaferrea
NA
g__UCG-005
g__uncultured
g__Family_XIII_UCG-001
g__Blautia
g__Ruminococcus
g__Angelakisella
g__UCG-005
g__Abiotrophia
g__Porphyromonas
g__[Eubacterium]_oxidoreducens_group
g__Bilophila
g__[Ruminococcus]_gauvreauii_group
g__Fusicatenibacter
g__Lactobacillus
g__Dorea
g__uncultured
g__TM7x
g__UCG-005
g__Anaerostipes
g__UCG-005
g__Christensenellaceae_R-7_group
g__UCG-005
g__Escherichia-Shigella
g__Saccharimonadaceae
g__Escherichia-Shigella
g__[Ruminococcus]_gauvreauii_group
g__Actinomyces
g__Butyricicoccus
asv
0.00 0.05 0.10 0.15 0.20
comp1
g__UCG-005
g__Bacteroides
g__Clostridia_vadinBB60_group
g__Butyricimonas
g__Sarcina
g__Barnesiella
g__Alistipes
g__Bacteroides
g__UCG-002
g__Lachnospiraceae_NK4B4_group
g__uncultured
g__UCG-010
g__UCG-010
g__Flavobacterium
NA
g__Muribaculaceae
NA
g__uncultured
g__Vibrio
g__Monoglobus
g__UCG-005
g__Ruminococcus
g__Clostridia_vadinBB60_group
g__Defluviitaleaceae_UCG-011
g__UCG-010
g__Sarcina
g__Prevotella
g__Alloprevotella
g__Lysinibacillus
g__Fusobacterium
g__Enterorhabdus
g__Candidatus_Bacilloplasma
g__uncultured
g__Lachnospiraceae_NK4A136_group
g__Dinghuibacter
g__Ruminococcus
g__UCG-010
g__Parabacteroides
g__Sanguibacteroides
g__Bacteroides
asv
-0.15 -0.10 -0.05 0.00
comp2
During nCCRT
g__Alloprevotella
g__RF39
g__Lachnoanaerobaculum
g__[Eubacterium]_coprostanoligenes_group
g__Leptotrichia
g__RF39
g__Christensenellaceae_R-7_group
g__Alistipes
g__Prevotella
g__Christensenellaceae_R-7_group
g__Johnsonella
g__Defluviitaleaceae_UCG-011
g__Prevotella
g__UCG-010
g__Treponema
g__Oribacterium
g__Fretibacterium
g__Dialister
g__RF39
NA
g__Dialister
g__uncultured
g__UCG-010
g__vadinBE97
g__Desulfovibrio
g__Fretibacterium
g__Prevotella
g__Colidextribacter
g__Leptotrichia
g__[Eubacterium]_coprostanoligenes_group
g__Peptococcus
g__Cloacibacillus
g__UCG-005
g__UCG-009
g__Treponema
g__Clostridia_UCG-014
g__Selenomonas
g__Johnsonella
g__Odoribacter
g__Peptoanaerobacter
g__UCG-010
g__Ruminococcus
g__Lentimicrobium
g__Family_XIII_UCG-001
g__Pseudomonas
g__Tuzzerella
g__Family_XIII_AD3011_group
g__RF39
g__[Eubacterium]_nodatum_group
g__Prevotella
g__Odoribacter
g__Alistipes
g__Faecalibaculum
g__Porphyromonas
asv
-0.20 -0.15 -0.10 -0.05 0.00
comp1
g__Sarcina
g__Lachnospiraceae_NK4A136_group
g__UCG-005
g__Roseburia
g__Christensenellaceae_R-7_group
g__Sanguibacteroides
g__UCG-008
g__Faecalibacterium
g__[Eubacterium]_coprostanoligenes_group
g__Christensenellaceae_R-7_group
g__UCG-010
g__Lachnospiraceae_NK4A136_group
g__Bacteroides
g__UCG-010
NA
g__Monoglobus
g__Terrisporobacter
g__Clostridia_vadinBB60_group
g__Lachnospiraceae_NK4A136_group
g__Ruminococcus
g__Desulfovibrio
NA
g__Bacteroidetes_vadinHA17
g__Lachnospiraceae_NK4B4_group
g__Sarcina
g__uncultured
g__Clostridia_vadinBB60_group
g__UCG-008
g__Lachnoclostridium
g__Veillonella
g__Odoribacter
g__Clostridia_vadinBB60_group
g__Oscillospira
g__Enterorhabdus
g__UCG-010
NA
g__Vicinamibacteraceae
g__uncultured
NA
g__Sphingomonas
g__DTU014
g__Butyrivibrio
g__Lachnospiraceae_NK4A136_group
g__Anaerofruncus
g__Bacteroides
g__Sarcina
g__Dysgonomonas
g__Barnesiella
g__Faecalibacterium
g__Blautia
g__Lachnoclostridium
g__Bacteroides
g__Christensenellaceae_R-7_group
g__UCG-010
g__Porphyromonas
g__RF39
asv
-0.15 -0.10 -0.05 0.00
comp2
After nCCRT
g__Lachnospiraceae_UCG-010
g__Ruminococcus
g__Prevotellaceae_UCG-004
g__Prevotella
g__Phascolarctobacterium
g__Prevotellaceae_Ga6A1_group
g__Prevotella
g__Holdemanella
g__Prevotella
g__Prevotellaceae_Ga6A1_group
g__Prevotella
g__Prevotellaceae_Ga6A1_group
g__Delftia
g__Holdemanella
g__Sutterella
g__Phascolarctobacterium
g__Megasphaera
g__Roseburia
g__Prevotella
g__Prevotellaceae_Ga6A1_group
g__Prevotella
NA
g__Collinsella
g__Lachnospira
asv
-0.20 -0.15 -0.10 -0.05 0.00
comp1
g__Coprobacter
g__Prevotella
g__Exiguobacterium
NA
g__Deinococcus
g__Clostridia_UCG-014
g__Sphingomonas
g__Lachnospiraceae_UCG-008
g__uncultured
g__Ellin6067
g__ZOR0006
g__Muribaculaceae
g__Paeniclostridium
g__MND1
g__Dongia
g__Bacteroides
g__Odoribacter
g__Muribaculaceae
g__Thermomonas
NA
g__Fusobacterium
g__Parabacteroides
asv
-0.2 -0.1 0.0 0.1
comp2

FigureS8

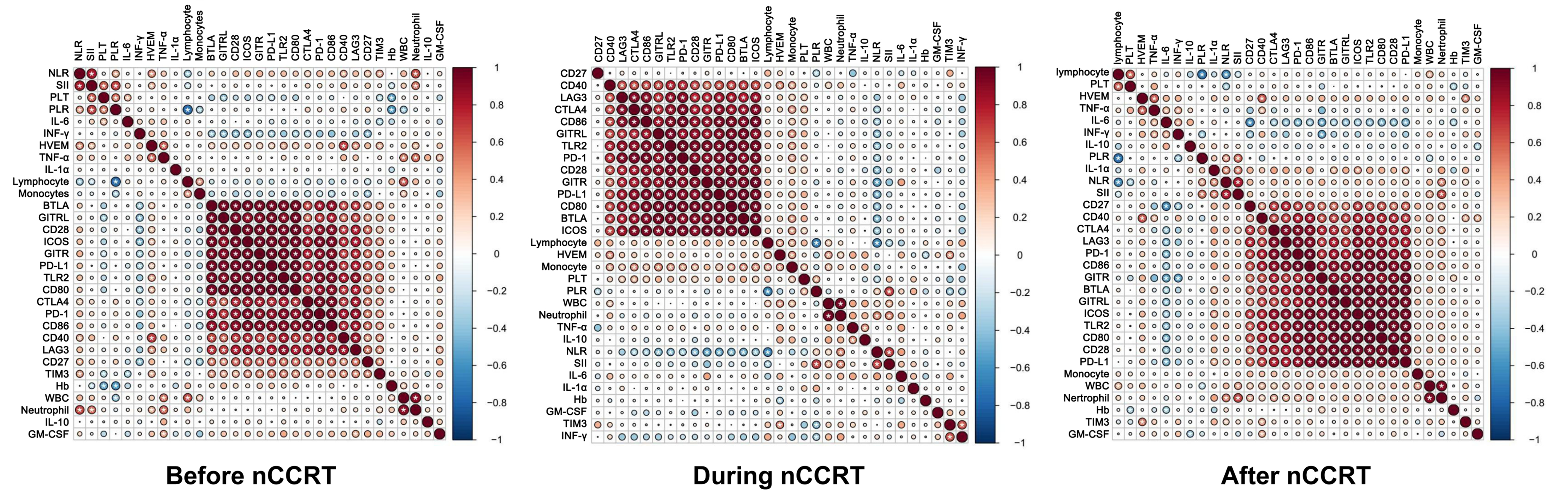
Before nCCRT
NLR SII PLT PLR IL-6 INF-γ HVEM TNF-α IL-1α Lymphocyte Monocytes BTLA GITRL CD28 ICOS GITR PD-L1 TLR2 CD80 CTLA4 PD-1 CD86 CD40 LAG3 CD27 TIM3 Hb WBC Neutrophil IL-10 GM-CSF
During nCCRT
CD27 CD40 LAG3 CTLA4 CD86 GITRL TLR2 PD-1 CD28 GITR PD-L1 CD80 BTLA ICOS Lymphocyte HVEM Monocyte PLT PLR WBC Neutrophil TNF-α IL-10 NLR SII IL-6 IL-1α Hb GM-CSF TIM3 INF-γ
After nCCRT
lymphocyte PLT HVEM TNF-α IL-6 INF-γ IL-10 PLR IL-1α NLR SII CD27 CD40 CTLA4 LAG3 PD-1 CD86 GITR BTLA GITRL ICOS TLR2 CD80 CD28 PD-L1 Monocyte WBC Nertrophil Hb TIM3 GM-CSF
1
0.8
0.6
0.4
0.2
0
−0.2
−0.4
−0.6
−0.8
−1

FigureS9
