# Supplementary material for: Prospective, longitudinal analysis of the gut microbiome in patients with locally advanced rectal cancer predicts response to neoadjuvant concurrent chemoradiotherapy
Source: J Transl Med. 2023 Mar 26;21:221. doi: 10.1186/s12967-023-04054-1 (PMC10041716; doi:10.1186/s12967-023-04054-1)
Supplement: Supplementary file 2 — Additional file 2: Table S1. Taxa identified by ZIBR as differing between the good- and poor-response groups. Table S2. Numbers of samples collected from patients before, during, and after neoadjuvant concurrent chemoradiation therapy. [file 12967_2023_4054_MOESM2_ESM.docx]

**Table S1**. Taxa identified by ZIBR as differing between the good- and poor-response groups.

| \|  \| Logistic Regression \| \| Beta Regression \| \|  \| \| --- \| --- \| --- \| --- \| --- \| --- \| \|  \| Coefficient \| *P* \| Coefficient \| *P* \| Joint P \| \| *Intestinimonas* \| 0.956 \| 0.319 \| 2.981 \| <0.001 \| <0.001 \| \| *Clostridium sensu_stricto_1* \| –2.082 \| 0.0041 \| –0.144 \| 0.688 \| 0.016 \| \| *[Ruminococcus] gauvreauii group* \| –1894.91 \| 0.088 \| –0.642 \| 0.025 \| 0.019 \| \| *UCG-010* \| –0.216 \| 0.891 \| –1.113 \| 0.006 \| 0.021 \| \| *Fusobacterium* \| 2.735 \| 0.007 \| –0.095 \| 0.759 \| 0.026 \| \| *Psychrobacter* \| –0.340 \| 0.749 \| 2.263 \| 0.005 \| 0.018 \| \| *Alloprevotella* \| 1.578 \| 0.030 \| 0.260 \| 0.606 \| 0.034 \| \| *Tyzzerella* \| –1.573 \| 0.029 \| 0.574 \| 0.189 \| 0.035 \| \| *Lachnospiraceae UCG-008* \| –1.738 \| 0.035 \| –1.866 \| 0.001 \| 0.008 \| \| *Ralstonia* \| 2.149 \| 0.023 \| –0.272 \| 0.477 \| 0.039 \| \| *Lachnospiraceae UCG-001* \| –1.064 \| 0.472 \| –1.433 \| 0.011 \| 0.005 \| |
| --- | --- | --- | --- | --- | --- | --- | --- | --- | --- | --- | --- | --- | --- | --- | --- | --- | --- | --- | --- | --- | --- | --- | --- | --- | --- | --- | --- | --- | --- | --- | --- | --- | --- | --- | --- | --- | --- | --- | --- | --- | --- | --- | --- | --- | --- | --- | --- | --- | --- | --- | --- | --- | --- | --- | --- | --- | --- | --- | --- | --- | --- | --- | --- | --- | --- | --- | --- | --- | --- | --- | --- | --- | --- | --- | --- | --- | --- | --- |

ZIBR, zero-inflated beta regression model with random effects

**Table S2.** Numbers of samples collected from patients before, during, and after neoadjuvant concurrent chemoradiation therapy.

| Sample | Before nCCRT | During nCCRT | After  nCCRT |
| --- | --- | --- | --- |
| 1 | 1 | 1 |  |
| 2 | 1 | 1 | 1 |
| 4 | 1 |  | 1 |
| 6 | 1 | 1 |  |
| 7 | 1 | 1 | 1 |
| 9 | 1 |  | 1 |
| 10 | 1 | 1 | 1 |
| 13 |  | 1 | 1 |
| 16 | 1 | 1 | 1 |
| 17 | 1 | 1 | 1 |
| 23 | 1 | 1 | 1 |
| 26 | 1 |  |  |
| 27 | 1 | 1 | 1 |
| 29 | 1 | 1 | 1 |
| 30 | 1 | 1 | 1 |
| 31 | 1 | 1 | 1 |
| 35 | 1 |  |  |
| 36 | 1 |  | 1 |
| 38 | 1 |  | 1 |
| 41 | 1 | 1 | 1 |
| 42 | 1 | 1 | 1 |
| 8 | 1 | 1 | 1 |
| 11 | 1 | 1 | 1 |
| 14 | 1 | 1 | 1 |
| 18 | 1 | 1 | 1 |
| 19 | 1 | 1 | 1 |
| 20 | 1 | 1 | 1 |
| 21 |  | 1 | 1 |
| 22 | 1 | 1 | 1 |
| 24 | 1 | 1 | 1 |
| 25 | 1 | 1 |  |
| 28 | 1 | 1 | 1 |
| 32 | 1 | 1 | 1 |
| 33 | 1 | 1 | 1 |
| 34 | 1 |  | 1 |
| 37 |  | 1 | 1 |
| 39 | 1 | 1 | 1 |
| 40 | 1 | 1 |  |
| sum | 35 | 31 | 32 |
